# Toward diagnostic relevance of the α_V_β_5_, α_V_β_3_, and α_V_β_6_ integrins in OA: expression within human cartilage and spinal osteophytes

**DOI:** 10.1038/s41413-020-00110-4

**Published:** 2020-09-30

**Authors:** Edith Charlier, Céline Deroyer, Sophie Neuville, Zelda Plener, Olivier Malaise, Federica Ciregia, Philippe Gillet, Gilles Reuter, Mallory Salvé, Nadia Withofs, Roland Hustinx, Dominique de Seny, Michel G. Malaise

**Affiliations:** 1 Laboratory of Rheumatology, GIGA-I3, CHULiège, ULiège, Liège, Belgium; 2 Department of Orthopaedic Surgery, CHULiège, Liège, Belgium; 3 Department of Neurosurgery, CHULiège, Liège, Belgium; 4 Department of Nuclear Medicine, CHULiège, Liège, Belgium

**Keywords:** Bone, Pathogenesis

## Abstract

We previously reported ^18^FPRGD_2_ uptake by the coxofemoral lining, intervertebral discs and facet joint osteophytes in OA using PET/SCAN imaging. However, the molecular mechanism by which the PRGD_2_ tracer interacts with joint tissues and osteophytes in OA remains unclear. As PRGD_2_ ligands are expected to belong to the RGD-specific integrin family, the purpose of this study was (i) to determine which integrin complexes display the highest affinity for PRGD2-based ligands, (ii) to analyze integrin expression in relevant tissues, and (iii) to test integrin regulation in chondrocytes using OA-related stimuli to increase the levels of fibrosis and ossification markers. To this end, the affinity of PRGD_2_-based ligands for five heterodimeric integrins was measured by competition with ^125^I-echistatin. In situ analyses were performed in human normal vs. OA cartilage and spinal osteophytes. Osteophytes were characterized by (immuno-)histological staining. Integrin subunit expression was tested in chondrocytes undergoing dedifferentiation, osteogenic differentiation, and inflammatory stimulation. The integrins α_V_β_5_, α_V_β_3_, and α_V_β_6_ presented the highest affinity for PRGD_2_-based ligands. In situ, the expression of these integrins was significantly increased in OA compared to normal cartilage. Within osteophytes, the mean integrin expression score was significantly higher in blood vessels, fibrous areas, and cells from the bone lining than in osteocytes and cartilaginous zones. In vitro, the levels of integrin subunits were significantly increased during chondrocyte dedifferentiation (except for β_6_), fibrosis, and osteogenic differentiation as well as under inflammatory stimuli. In conclusion, anatomical zones (such as OA cartilage, intervertebral discs, and facet joint osteophytes) previously reported to show PRGD_2_ ligand uptake in vivo expressed increased levels of α_V_β_5_, α_V_β_3_, and β_6_ integrins, whose subunits are modulated in vitro by OA-associated conditions that increase fibrosis, inflammation, and osteogenic differentiation. These results suggest that the increased levels of integrins in OA compared to normal tissues favor PRGD2 uptake and might explain the molecular mechanism of OA imaging using the PRGD_2_-based ligand PET/CT.

## Introduction

According to estimates published in 2016, up to 240 million people around the world suffer from osteoarthritis (OA).^1^ It is the most common chronic joint disease in adults and is characterized by joint pain, short-lived morning stiffness, and functional limitations. OA generally affects one joint, especially the knee or hip but also frequently the hand or spine. Age and obesity have been identified as prominent risk factors for OA development, although the precise causes are still unknown. Medical treatment consists of pain reduction and improvement of physical function and quality of life, but to date, the course of OA remains unstoppable, and the treatment of last resort consists of prosthetic surgery.^2^

Within the OA joint, several anatomic changes occur, such as synovial hyperplasia, subchondral bone sclerosis, cartilage erosion, and osteophyte formation. Among these alterations, osteophyte formation and joint space narrowing (due to cartilage erosion) constitute two major radiographic criteria for OA diagnosis according to the gold standard Kellgren–Lawrence grading system.^3,4^ However, although conventional radiography is widely used for OA diagnosis,^5^ it is not sensitive enough to detect early degenerative changes in cartilage, and it allows only a static view of the disease. Indeed, the disease frequently progresses for months or even years before a diagnosis is made. Therefore, functional imaging of OA is awaited in clinical practice to probe tissue function and better evaluate disease progression.^4^ The positron emission tomography (PET) imaging method might fulfill these expectations while also providing quantitative information. To support this idea, we previously reported observations of OA joint uptake of the ^18^F-FPRGD_2_ radioligand in PET/CT images, suggesting the possibility of visualizing the OA process in vivo.^6^ The main OA-related structures highlighted by the tracer were the coxofemoral lining (identified as osteoarthritic by concomitant CT), the osteophytes next to facet joints and the osteophytes on the margins of vertebral bodies next to degenerative discs.

Cartilage is composed of only one cell type, chondrocytes, which are wrapped in a collagen-rich extracellular matrix that they synthesize. Compared to normal cartilage, OA cartilage is characterized by the perturbation of chondrocyte viability^7^ and the secretory profile.^8^ The acquisition of a hypertrophic phenotype (with production of aberrant type X collagen and catabolic MMP-13 protease) by chondrocytes is well documented and contributes to matrix calcification and OA development.^9^ However, it is increasingly believed that chondrocytes acquire a variety of degenerative phenotypes during the course of OA,^10^ including that of the “fibro-chondrocyte”, which secretes type III collagen that might be involved in the increased fibrosis observed within the OA cartilage matrix.^11,12^

Osteophytes or osteochondrophytes^13^ consist of neoplastic cartilagino-osseous protrusions at the margins of OA diarthrodial joints^14^ or at the spine level.^15^ Depending on their location but also on the stage of the disease, their formation in OA joints has been described either (i) as a functional adaptation to cope with OA-associated joint instability^15,16^ or (ii) as a pathological structure per se.^13^ In particular, osteophyte formation is associated with knee deformity^16^ and knee pain;^17^ depending on the spine location (cervical, thoracic, or lumbar), osteophytes can compress the esophagi, nerves or arteries,^18^ leading to severe pain,^19^ dysphagia,^20^ vocal cord paralysis^21^ and compromised breathing^22^ (according to a review^15^).

The molecular target of the ^18^F-FPRGD_2_ radiopharmaceutical compound used for PET/CT imaging is reported to be α_v_β_3_ integrin,^23^ although other RGD-specific integrins (with the arginine–glycine–asparagine sequence) might also interact. Integrins are noncovalently associated α/β heterodimeric transmembrane receptors that control various physiological processes, ranging from cell adhesion^24,25^ and mechanosensing^26^ to proliferation and differentiation.^27,28^ Among the 24 known integrin complexes, 8 are receptors for proteins containing an RGD motif. Three of them (α_V_β_3_, α_V_β_5_, and α_5_β_1_) recognize major RGD-containing matrix components such as vitronectin (α_V_β_5_ and α_V_β_3_), fibronectin (α_5_β_1_), or osteopontin (α_V_β_3_).^27^ In situ, α_V_β_5_, α_V_β_3_, and α_5_β_1_ are expressed by chondrocytes within human normal knee cartilage sections.^29^ The comparison of RGD-specific integrin expression between normal and OA cartilage has not been well explored in situ. One study reported that the α_1_, α_5_, α_V_, β_1_, β_4_, and β_5_ integrin subunits were expressed by both normal and OA femoral cartilage, whereas the α_2_, α_4_, and β_2_ subunits were restricted to OA femoral cartilage.^30^ In a previous work, we identified increased expression of α_V_β_3_ in OA femoral cartilage compared with normal femoral cartilage.^6^ In vitro, α_V_, α_5_, β_1_, and α_V_β_5_ were detected at the cell surface of chondrocytes freshly isolated from normal knee cartilage by flow cytometry.^29^ In OA knee cartilage, 40% of isolated chondrocytes expressed the β_1_ integrin subunit at their cell surface as well as all its α subunit binding partners.^31^ Interestingly, we found differential integrin expression in human femoral OA chondrocytes during monolayer culture-induced dedifferentiation, as α_v_, β_3_, and α_v_β_3_ were detected in dedifferentiated OA fibrochondrocytes but not in freshly isolated OA chondrocytes.^6^

In this work, we addressed the relevance of RGD-specific integrins as molecular targets for PRGD_2_ ligands and therefore as potential markers for OA diagnosis. First, we examined which RGD-specific integrins exhibited the highest affinity for PRGD_2_-based ligands. Second, we tested their expression in situ in PRGD_2_-accumulating tissues, namely, human OA cartilage and spinal osteophytes (from the facet joint and the degenerative disc). Finally, we investigated integrin subunit regulation in chondrocytes in vitro in the OA-related contexts of chondrocyte dedifferentiation, fibrosis, inflammation, and osteogenic differentiation (via increases in mineralization markers).

## Results

### PRGD2 uptake by OA structures

**Fig. 1:**
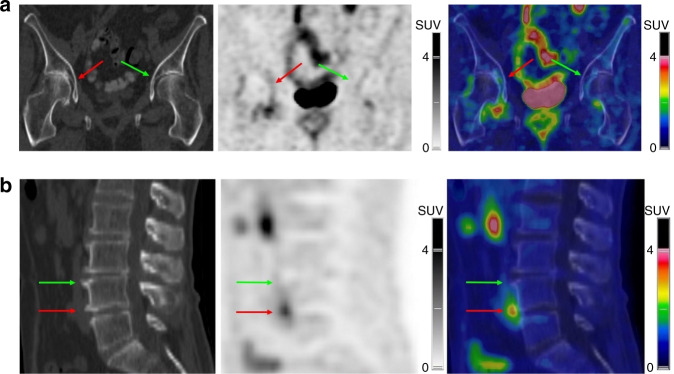
Location of ^18^F-FPRGD2 uptake. ^18^F-FPRGD2 PET/CT images (A: CT; B: PET; C: fused PET/CT) from an 80-year-old patient with bilateral coxofemoral osteoarthritis. ^18^F-FPRGD2 uptake is higher in the right joint, including the region of articular cartilage identified by the red arrows (SUVmax 2.3 target SUVmax-to-muscle SUVmax ratio 3.1). Based on CT images, degenerative changes are also more advanced in the right coxofemoral joint (**a**). ^18^F-FPRGD_2_ PET/CT images (A: CT; B: PET; C: fused PET/CT) of a 74-year-old patient showing osteophytes at the anterior margins of the L3–L4 disc (pointed to by green arrows) and L4–L5 disc (pointed to by red arrows), yet only the latter showed high ^18^F-FPRGD_2_ uptake (maximum standardized uptake value SUV_max_ 3.2; target SUV_max_-to-muscle SUV_max_ ratio 6.5) (**b**)

We previously reported that the radiolabeled ligand ^18^F-FPRGD_2_ was taken up by hip OA structures^6^ identified as osteoarthritic by CT (Fig. 1a left, green and red arrows). One OA structure showed intense focal uptake of ^18^F-FPRGD_2_ (Fig. 1a middle and right, red arrow). In the same way, Fig. 1b shows osteophyte structures at the anterior margins of the L3–L4 disc (green arrows) and L4–L5 disc (red arrows). Only the osteophytes at the L4–L5 disc showed high ^18^F-FPRGD_2_ uptake (red arrows).

### Integrin binding specificity of PRGD_2_ ligands

The initial study reporting tracer uptake by OA structures used the radiolabeled ligand ^18^F-FPRGD_2_.^6^ According to its RGD sequence, this ligand could potentially interact with eight members of the RGD-specific integrin family. Therefore, the first goal of this work was to characterize PRGD_2_ ligand affinity for a panel of RGD-specific integrins. For future clinical applications, another radioisotope (i.e., ^68^Ga) could be considered to simplify the PRGD_2_ radiolabeling process.^32^ During this process, ^68^Ga complexation can be achieved either with NOTA or NODAGA chelators, which are known to have different pharmacokinetic properties toward radioligands in vivo.^32^ Therefore, we included the characterization of NOTA-PRGD_2_ and NODAGA-PRGD_2_ in this study to test whether these chelators could affect PRGD_2_ affinity for integrins and thereby guide future chelator selection. The chemical structures of these ligands are presented in Fig. S1.

**Fig. 2:**
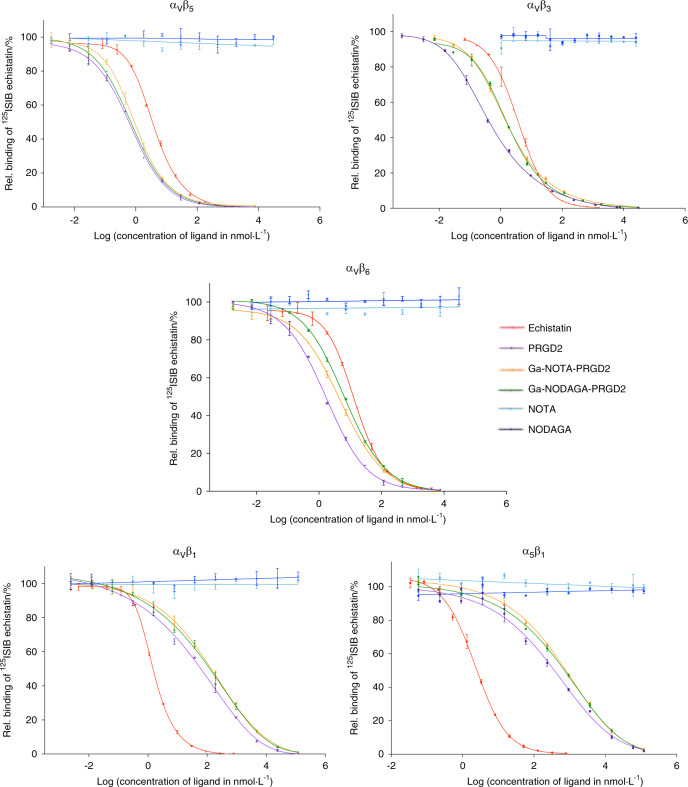
Competition studies between ^125^I-echistatin and PRGD_2_ ligands for surface-immobilized integrins. To determine PRGD_2_ affinity for selective integrins, ^125^I-labeled echistatin and variable concentrations of unlabeled PRGD_2_ ligands were added to microtiter plates coated with detergent-solubilized integrin receptor subtypes α_v_β_5_, α_v_β_3_, α_v_β_6_, α_v_β_1_, and α_5_β_1_. For each ligand concentration, the relative binding of ^125^I-echistatin was then calculated, and the values were plotted against the logarithm of ligand concentration. The experimental data were subjected to nonlinear regression using a five-parameter logistical model with GraphPrism 5 software. They are presented as an asymmetrical (five-parameter) logistic dose-response curve. Measurements were performed in duplicate. Unlabeled echistatin, which binds to all five integrins with high affinity, was used as a positive control (red curves). NOTA and NODAGA chelators alone were used as negative controls (light and dark blue lines). The corresponding IC_50 values_ are listed in Table 1

Ligand affinity characterization was performed by competition binding assays between ^125^I-SIB-echistatin (i.e., a potent integrin antagonist) and PRGD_2_-based ligands for precoated integrin complexes (i.e., α_V_β_5_, α_V_β_3_, α_V_β_6_, α_V_β_1_, and α_5_β_1_). We tested five RGD-specific integrins out of eight members of the family based on the reported cellular distribution of integrins in vitro.^27^ For each complex, the effect of different concentrations of PRGD_2_ ligands on ^125^I-SIB-echistatin binding was examined, and displacement curves were generated (Fig. 2). From these, the IC_50_ values were calculated (Table 1). The IC_50_ of a ligand represents the concentration of ligand required to displace 50% of ^125^I-echistatin from the integrin complex. Unlabeled echistatin was used as a positive control and bound to all of the tested integrins, which was consistent with previous reports (Fig. 2, red curve on the graphs).^33^ NOTA and NODAGA chelators alone were used as negative controls, and as expected, they did not bind to any of the integrins tested (Fig. 2, light and dark blue lines on the graphs, respectively). All three PRGD_2_-based ligands bound within a subnanomolar range to α_V_β_5_ and within a nanomolar range to α_V_β_3_ and α_V_β_6_ (Table 1). Moreover, for these three integrin complexes, the IC_50_ of all the PRGD_2_ ligands was always inferior to the IC_50_ of unlabeled echistatin, suggesting a strong interaction (Fig. 2, violet, yellow and green curves at the left of the red curve). By contrast, α_V_β_1_ and α_5_β_1_ bound the PRGD_2_ ligands within the submicromolar range (Fig. 2, violet, yellow and green curves to the right of the red curve). In conclusion, it is likely that the complexes recognized in vivo by PRGD_2_-based ligands bind in the order α_V_β_5_, α_V_β_3_, and α_V_β_6_ regardless of NOTA or NODAGA binding.

**Table 1:** IC50 of competition studies between ^125^I-echistatin and PRGD_2_ ligands for surface-immobilized integrins

IC_50_ values (nmol·L^−1^) of ligands for coated RGD-specific rh integrin complexes					
Ligands	RGD-specific integrin complexes				
	α_V_β_5_	α_V_β_3_	α_V_β_6_	α_V_β_1_	α_5_β_1_
NOTA-PRGD_2_	0.94	1.53	4.93	148.36	674.87
NODAGA-PRGD_2_	0.69	1.74	6.32	88.88	666.15
PRGD_2_	0.65	0.45	1.61	42.85	369.39
Echistatin	3.92	3.69	13.85	1.54	2.24

To determine PRGD_2_ IC_50_ for selective integrins, ^125^I-labeled echistatin and variable concentrations of unlabeled PRGD_2_ ligands were added to microtiter plates coated with detergent-solubilized integrin receptor subtypes α_v_β_5_, α_v_β_3_, α_v_β_6_, α_v_β_1_, and α_5_β_1_. IC_50_ values (nmol·L^−1^) from competition binding assays are summarized. They were obtained from competition curves (Fig. 2) and determined by GraphPad Prism software

### Expression of α_V_β_5_, α_V_β_3_, and β_6_ in human femoral cartilage

To increase the relevance of PET/CT using PRGD_2_-based ligands, we tested whether anatomical OA structures showing ^18^F-FPRGD_2_ uptake in vivo effectively expressed α_V_β_5_, α_V_β_3_, and α_V_β_6_ in situ. We performed immunohistochemical detection in femoral cartilage and vertebral osteophyte sections using antibodies directed against a specific integrin heterodimer (i.e., α_V_β_5_ or α_V_β_3_). As antibodies recognizing the heterodimer α_V_β_6_ are not recommended for IHC-paraffin application, we used an antibody directed against the β_6_ subunit.

**Fig. 3:**
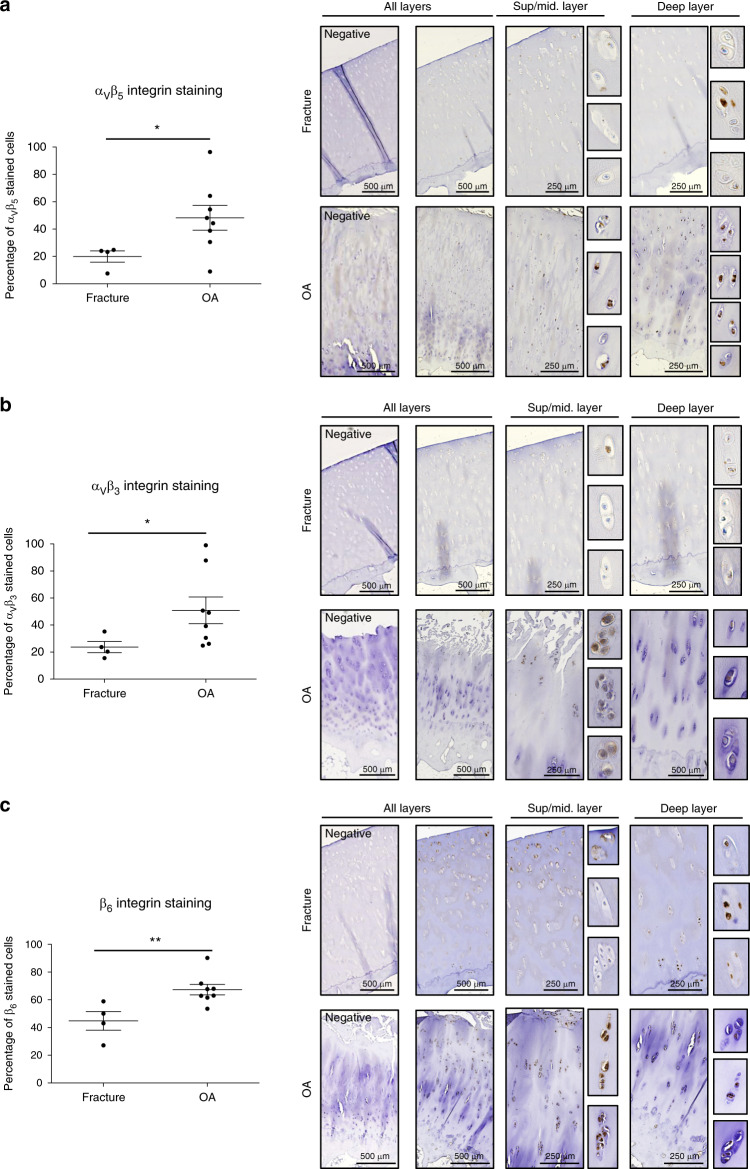
Integrin expression within healthy (fracture) and OA human cartilage. Representative IHC pictures of human cartilage sections from hip fracture (upper panel) or OA hip (lower panel) stained with **a** anti-α_v_β_5_, **b** anti-α_v_β_3_, or **c** anti-β_6_ antibodies. The negative control (primary antibody omitted) for each staining is presented on the left. Analysis was performed on several patients, and the percentage of positively stained cells was significantly increased in OA hips (*n* = 8) compared to hip fractures (*n* = 4) (Mann–Whitney *U* test for fracture vs. OA comparisons: α_v_β_5_, *P* = 0.028 3; α_v_β_3_, *P* = 0.028 3; β_6_, *P* = 0.008 1)

The IHC results obtained for the femoral cartilage samples are presented in Fig. 3. No background staining was observed when the primary antibody was omitted (negative). Integrin immunostaining was quantified, and the values in the graphs represent the percentages of positively stained chondrocytes in either a normal (fracture) or OA cartilage sample. The percentage of stained cells was significantly increased in OA cartilage compared to that in normal cartilage for α_V_β_5_ (normal: 19.9 ± 4.1 vs. OA: 48.2 ± 9.0, *P* = 0.028 3), α_V_β_3_ (normal: 23.7 ± 4.2 vs. OA: 50.9 ± 9.9, *P* = 0.028 3) and β_6_ (normal: 44.8 ± 6.7 vs. OA: 67.3 ± 3.8, *P* = 0.008 1) (Fig. 3a–c, respectively). Notably, in normal cartilage, integrin expression was evenly distributed among the three cartilage layers. In OA cartilage, expression was more important in the middle zone for the three integrins (Fig. S2a).

Interestingly, for the expression of α_5_β_1_ (i.e., the integrin presenting a low affinity for PRGD_2_ ligands), we did not observe any significant difference between normal and OA cartilage (normal: 48.6 ± 6.0 vs. OA: 51.5 ± 8.2, *P* = 0.933 3) (Fig. S2b). In conclusion, the α_V_β_5_, α_V_β_3_, and α_V_β_6_ integrins, which present the highest affinity for PRGD_2_ ligands, are overexpressed in OA cartilage compared to normal cartilage, suggesting that they may be able to discriminate OA cartilage, in contrast to α_5_β_1_.

### Histological characterization of human spinal osteophytes

Osteophytes from OA facet joints and degenerative discs were first characterized at the histological level according to Junker’s classification, which was previously established for knee osteophytes.^34^ Based on the relative proportions of bone, cartilage, and mesenchymal connective tissue (mCT), the authors identified four different osteophyte types: type A (consisting solely of mCT and cartilage), type B (containing mCT, cartilage and less than 20% ossified areas), type C (containing cartilage, >10% mCT and >20% ossified areas), and type D (containing cartilage, <10% mCT and >20% ossified areas).^34^

Accordingly, we performed alizarin red staining and type II collagen immunostaining to visualize bone and cartilage surfaces. For each osteophyte sample (35 samples, 9 patients), the total, bone, and cartilage outlines were determined according to the staining (Fig. S3), and the resulting areas were calculated using Cytomine software (Table 2). The mCT surface was determined by subtracting the bone and cartilage area from the total area (Fig. S3) and was stained green by conventional Masson trichrome staining (data not shown). By calculating the percentages of bone and mCT areas for each sample, we could rank osteophytes according to Junker’s classification (Table 2). We found mostly type C (21/35, 60%) and type D (9/35, 25.7%) osteophytes, whereas type B (4/35, 11.4%) and type A (1/35, 2.9%) osteophytes were less well represented. Representative pictures of each osteophyte type, with the stained and drawn areas, are presented in Fig. S3. The mean surface area of spinal osteophytes ranged from 8.9 to 103.1 mm^2^, with a mean of 29.3 mm^2^ (see Table 2). The bone area ranged from 0.03 to 74.7 mm^2^ with a mean of 14.5 mm^2^; the cartilage area ranged from 0.1 to 20.5 mm^2^ with a mean of 11.3 mm^2^; and the mCT area ranged from 0.4 to 22.5 mm^2^ with a mean of 10.0 mm^2^.

**Table 2:** Histological characterization of human spinal osteophytes samples

Samples #	Anatomic location	Osteophyte origin	Total area/mm²	Bone area/mm²	Cartilage/mm^2^	mCT/mm^2^	Bone/%	mCT/%	Classification^34^	(Cartilage + mCT) area				Bone area			
										Pure COL2	Pure COL3	Overlapping COL2 COL3	Blood vessels	Blood vessels	Bone marrow cells	Bone lining cells	Osteocytes
1	Cervical	DD	9.4	0.03	8.2	1.17	0.3	12.4	A	0	0	1	1	0	0	0	0
2	Cervical	DD	14.4	0.8	5.4	8.2	5.6	56.9	B	0	1	1	1	0	0	1	1
3	Cervical	DD	20.8	1.3	15.7	3.8	6.3	18.3	B	0	0	1	0	0	0	1	1
4	Lumbar	FJ	38.3	3.4	19.5	15.4	8.9	40.2	B	0	1	1	1	1	1	1	1
5	Lumbar	FJ	36.2	7	8.7	20.5	19.3	56.6	B	1	1	1	1	1	1	1	1
6	Lumbar	FJ	37.9	30.2	3.6	4.1	79.7	10.8	C	1	1	1	0	1	0	1	1
7	Lumbar	FJ	26.8	18.4	4.7	3.7	68.7	13.8	C	1	1	1	1	1	1	1	1
8	Cervical	DD	14.8	10.3	2.4	2.1	69.4	14.5	C	1	0	1	0	1	1	1	1
9	Cervical	DD	28.3	18.2	5.8	4.3	64.3	15.2	C	1	0	1	1	0	1	1	1
10	Lumbar	FJ	67.5	53.8	2	11.7	79.7	17.3	C	1	1	1	1	1	0	1	1
11	Lumbar	FJ	45.3	19.9	15.9	9.5	43.9	21.0	C	1	1	1	1	1	1	1	1
12	Lumbar	FJ	32.1	21.6	3.7	6.8	67.3	21.2	C	0	1	1	0	1	0	1	1
13	Lumbar	FJ	12	8.5	0.6	2.9	70.8	24.2	C	0	1	1	1	0	0	1	1
14	Lumbar	FJ	22.2	10.6	6.1	5.5	47.7	24.8	C	0	1	1	0	1	0	1	1
15	Lumbar	FJ	24.8	12.7	5.5	6.6	51.2	26.6	C	1	1	1	1	1	1	1	1
16	Cervical	DD	24	12.4	5.1	6.5	51.7	27.1	C	1	0	0	0	1	1	1	1
17	Lumbar	FJ	34.7	14.5	10.2	10	41.8	28.8	C	0	1	1	0	1	0	1	1
18	Lumbar	FJ	13	8.8	0.3	3.9	67.7	30.0	C	0	1	1	0	1	0	1	1
19	Lumbar	FJ	49	27.9	5.2	15.9	56.9	32.4	C	0	1	1	0	1	0	1	1
20	Cervical	DD	19.1	4.9	7.6	6.6	25.7	34.6	C	0	0	1	1	1	1	1	1
21	Lumbar	FJ	64.1	22	19.6	22.5	34.3	35.1	C	0	1	1	1	1	0	1	1
22	Lumbar	FJ	63	34.4	6.4	22.2	54.6	35.2	C	0	1	1	1	1	0	1	1
23	Cervical	DD	24.4	10.1	5.1	9.2	41.4	37.7	C	0	0	1	1	0	1	1	1
24	Lumbar	FJ	23.3	7.3	4.8	11.2	31.3	48.1	C	0	1	1	0	1	0	1	1
25	Lumbar	FJ	24.6	6.4	4.9	13.4	25.8	54.3	C	0	1	1	0	1	0	1	1
26	Cervical	DD	28.9	5.9	3.9	19.1	20.4	66.1	C	1	0	1	1	0	1	1	1
27	Lumbar	FJ	27.5	9.3	15.5	2.7	33.8	9.8	D	1	1	1	0	0	1	1	1
28	Cervical	DD	8.9	7.3	0.8	0.8	82.0	9.0	D	1	1	0	0	1	1	1	1
29	Cervical	DD	20.9	10.7	8.7	1.5	51.2	7.2	D	1	0	1	0	1	0	1	1
30	Lumbar	FJ	20.8	15.3	4	1.5	73.6	7.2	D	0	0	1	0	1	0	1	1
31	Lumbar	FJ	11.5	10.9	0.1	0.6	94.3	5.0	D	0	1	1	0	1	0	1	1
32	Cervical	DD	11.2	2.9	7.8	0.5	25.9	4.5	D	0	0	1	0	1	0	1	1
33	Lumbar	FJ	36.6	14.9	20.5	1.2	40.7	3.3	D	1	0	1	0	1	0	1	1
34	Lumbar	FJ	15	14.3	0.2	0.5	95.3	3.3	D	0	1	1	0	1	1	1	1
35	Lumbar	FJ	103.1	74.7	28	0.4	72.5	0.4	D	1	0	1	0	1	1	1	1

For each spinal osteophyte sample, the left part of the table presents the following characteristics: osteophyte vertebral location (cervical or lumbar), osteophyte origin (from facet joint, FJ or from degenerative disc, DD), the total, bone, and cartilage areas (in mm^2^, drawn and determined with Cytomine software), the mCT area (determined by subtraction of bone and cartilage areas from the total area: see Fig. S3), the percentage of bone and mCT areas allowing determination of the osteophyte type (A, B, C, or D, according to Junker’s classification established for knee osteophytes^34^). The right part of the table indicates the presence (1) or absence (0) of cartilage (pure COL2, areas positive for immunostaining with anti-type II collagen antibody), fibrous tissue (pure COL3, areas positive for immunostaining with anti-type III collagen antibody), fibrocartilage (overlapping COL2 COL3, overlapping areas positive for both type II and III collagen immunostaining), blood vessels and specific cell types such as bone marrow cells (BMCs), osteocytes and cells from the bone lining within each spinal osteophyte sample. Complementarily, a representative picture of each osteophyte type can be found in Fig. S3, as well as the way mCT areas were obtained. Scale bars are reported on each picture

In ossified areas, we identified two types of bone structure: (i) a cancellous bone type, with trabeculae (enclosing few osteocytes) separating fatty areas (Fig. S4a) and infiltration of bone marrow cells (BMCs), in 15/35 samples (42.9%) (Table 2); and (ii) a denser bone type, which was essentially composed of osteocytes. Combined bone types (trabecular and dense) were also observed (Fig. S4a). Osteocytes were either trapped in the lamellar parallel bone matrix or sparsely organized, as observed in woven bone (Fig. S4b). Bone lining cells (BLCs) were generally found in crown outlining fatty areas (Fig. S4c). A few osteoclasts could be identified based on their morphology, suggesting areas of bone remodeling (Fig. S4d). Finally, blood vessels were identified in 33/35 (94.3%) of the analyzed vertebral osteophytes and were located mostly within the mineralized area (27/35 samples, 77.1%) and/or the unmineralized area (15/35, 42.9%).

The unmineralized area of osteophytes (cartilage + mCT) appeared less heterogeneous than the mineralized area and consisted of cells dispersed in a matrix. The nature of this matrix was refined using immunohistological staining with anti-type II and anti-type III collagen antibodies. Type II collagen staining was considered a cartilage signature, whereas type III collagen staining identified fibrous areas. When type II and III staining overlapped, the zone was identified as fibrocartilage. WE detected fibrocartilage areas in 33/35 (94.3%) of the tested samples. In addition to fibrocartilage, samples could also include “pure” fibrous areas and/or “pure” cartilaginous areas (Table 2).

### Expression of α_V_β_5_, α_V_β_3_, and β_6_ in human spinal osteophytes

**Fig. 4:**
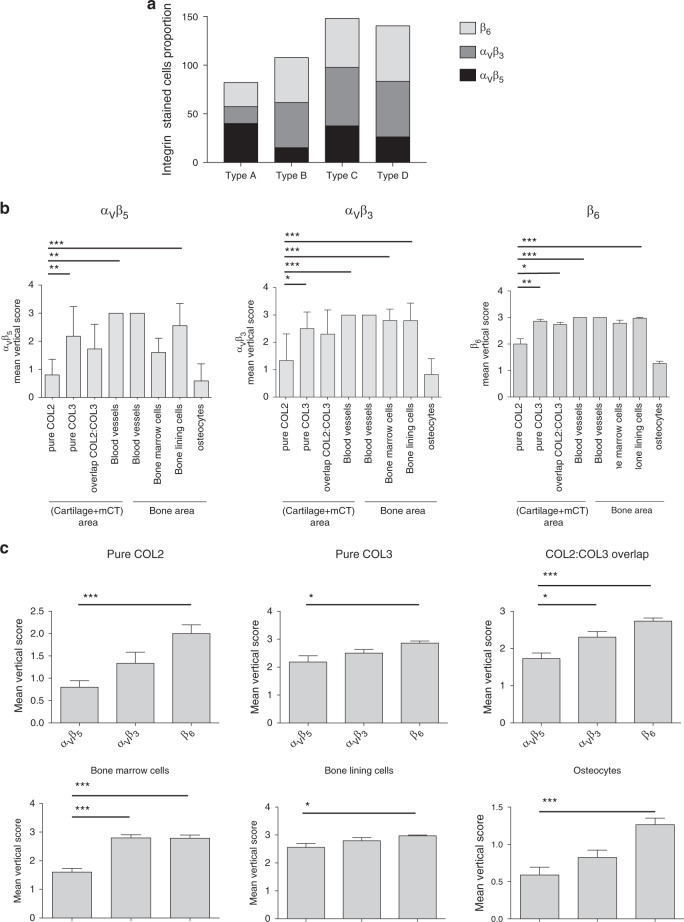
Statistical analyses of integrin expression scores within spinal osteophytes. **a** Graphical representation of integrin expression along osteophyte types (according to the Junker Classification: type A *n* = 1, type B *n* = 4 type C *n* = 21, and type D *n* = 9). The percentage of positively stained cells for each integrin is presented in black for α_V_β_5_, in dark gray for α_V_β_3_ and in light gray for β_6._ For each integrin, the mean vertical expression scores were calculated for each compartment (see last lines of Table S2 (α_v_β_5_), Table S3 (α_v_β_3_), and Table S4 (β_6)_). **b** For each integrin staining, the mean vertical expression scores obtained for each compartment/cell type were compared (*n* = 35). **c** For each compartment/cell type, the mean vertical expression score of each integrin was compared (*n* = 35). Statistical analyses were performed using one-way ANOVA (Kruskal–Wallis test), and Dunn’s multiple comparison test was used for comparisons between two groups

Integrin expression (percentage of positively stained cells) was first calculated for the whole surfaces of osteophytes. The cumulative value for all integrin expression was high for each osteophyte type: type A: 82, type B: 108, type C: 148, and type D: 140. Moreover, integrin expression was upregulated in the late stages of osteophyte formation (types C and D) (Fig. 4a). Integrin expression was further explored and scored within each compartment, and the cell type was identified (α_V_β_5_, Table S2, α_V_β_3_, Table S3, and β_6_, Table S4). A score of 0 was given when no cells were stained; a score of 1 indicated that <50% of cells were stained; a score of 2 indicated that >50% of cells were stained; and a score of 3 indicated that all cells were stained. For each integrin, the mean vertical expression score (i.e., the mean integrin expression score from each osteophyte sample for one compartment/cell type) obtained for each compartment was calculated for all 35 samples analyzed (see last line of Tables S2–S4).

**Fig. 5:**
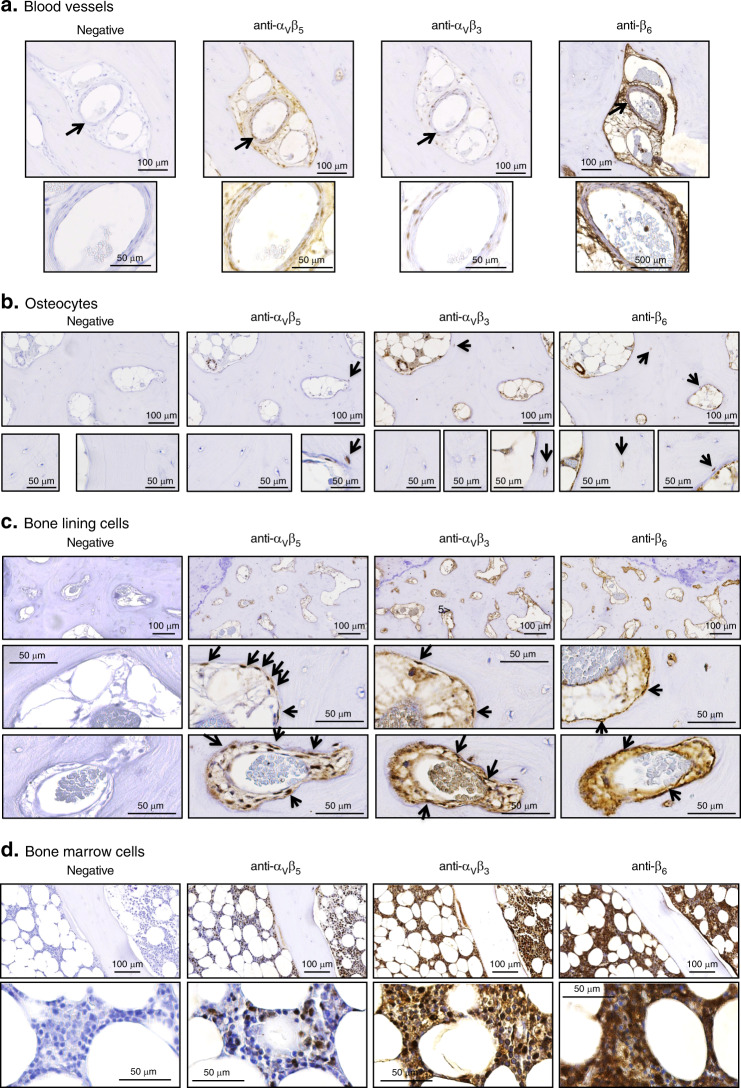
Integrin expression within blood vessels and bone compartments of spinal osteophytes. Representative pictures of spinal osteophyte sections either stained without primary antibody (negative) or with primary antibodies directed against α_V_β_5_, α_V_β_3_, or β_6_. Integrin expression was analyzed within **a** blood vessels (indicated by black arrows); expression score: for α_V_β_5_ = 3, for α_V_β_3_ = 3, and for β_6_ = 3; **b** osteocytes: only stained cells are indicated by black arrows; expression score: for α_V_β_5_ = 1, for α_V_β_3_ = 1, and for β_6_ = 1; **c** cells from the bone lining (BLC): two zones display organization in crown; expression score: for α_V_β_5_ = 3, for α_V_β_3_ = 3, and for β_6_ = 3; **d** bone marrow cells (BMCs); expression score: for α_V_β_5_ = 1, for α_V_β_3_ = 3, and for β_6_ = 3. Large pictures (×10), insert (×40). Scale bars are indicated on each picture

In bone areas, for the three integrins tested, the highest mean expression scores were attributed to blood vessels (=3 for α_V_β_5_ α_V_β_3_ and β_6_), whereas the lowest mean scores were attributed to osteocytes (α_V_β_5_ = 0.6 ± 0.13; α_V_β_3_ = 0.8 ± 0.11; and β_6_ = 1.3 ± 0.03) (Fig. 4b). Representative images of integrin staining within blood vessels and osteocytes are shown in Fig. 5a, b, respectively. In addition to blood vessels, cells from the bone lining were associated with higher expression scores for all integrins tested (α_V_β_5_ = 2.6 ± 0.1; α_V_β_3_ = 2.8 ± 0.1; and β_6_ = 2.9 ± 0.03), followed by BMCs (α_V_β_5_ = 1.6 ± 0.13; α_V_β_3_ = 2.8 ± 0.10; and β_6_ = 2.8 ± 0.11) (Fig. 4b). Representative images of integrin staining in cells from the bone lining and bone marrow are depicted in Fig. 5a, d, respectively.

**Fig. 6:**
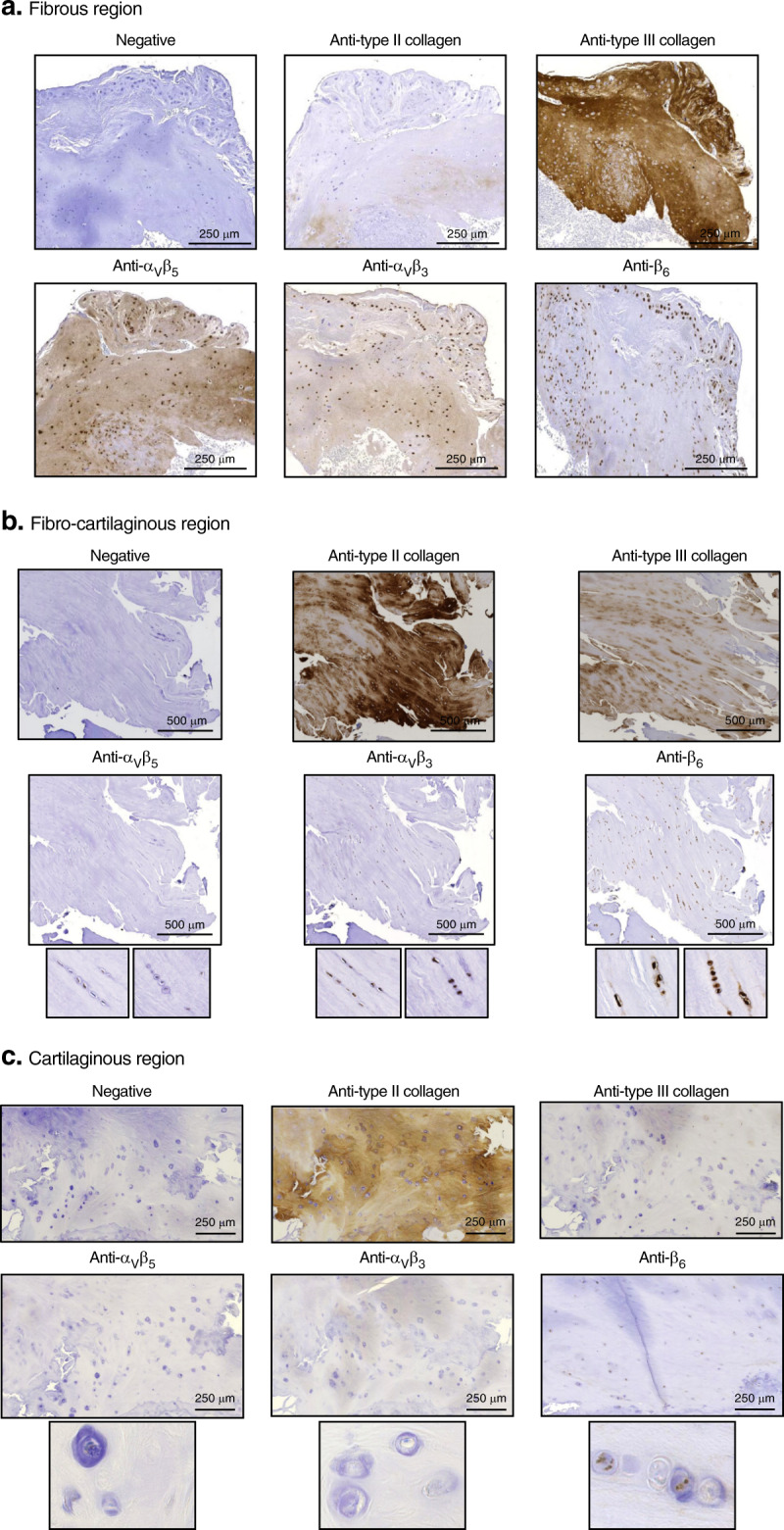
Integrin expression within (cartilage + mCT) compartments of spinal osteophytes. Representative pictures of spinal osteophyte sections stained either without primary antibody (negative) or with primary antibodies directed against type II collagen, type III collagen, α_V_β_5_, α_V_β_3_, or β_6._ Integrin expression was analyzed within **a** the fibrous region (positive for type III collagen); expression score: for α_V_β_5_ = 3, for α_V_β_3_ = 3, and for β_6_ = 3; **b** the fibrocartilaginous region (positive for both type II and type III collagen); **c** the cartilaginous region (positive for type II collagen); expression score for α_V_β_5_ = 1, for α_V_β_3_ = 0, for β_6_ = 2. Large pictures (×10), insert (×40). Scale bars are reported on each umage

In the cartilage + mCT area, the highest mean expression score for the three integrins tested was also attributed to blood vessels (=3 for α_V_β_5_ α_V_β_3_ and β_6_) (Fig. 4b). The fibrous zone (i.e., pure COL3) constitutes the second compartment of the cartilage + mCT area, where the mean expression score of the three integrins was the highest (α_V_β_5_ = 2.2 ± 0.20; α_V_β_3_ = 2.5 ± 0.13; and β_6_ = 2.9 ± 0.07), followed by the fibrocartilaginous area (i.e., pure COL3, COL2:COL3 overlap) (α_V_β_5_ = 1.7± 0.15; α_V_β_3_ = 2.3 ± 0.15; and β_6_ = 2.7 ± 0.08) (Fig. 4b). In contrast, the mean score obtained for the cartilaginous compartment (i.e., pure COL2) (α_V_β_5_ = 0.8 ± 0.14; α_V_β_3_ = 1.3 ± 0.25; and β_6_ = 2.0 ± 0.19) was significantly lower than that obtained for blood vessels, the fibrous zone, and cells from the bone lining for α_V_β_5_ and that obtained for BMCs for α_V_β_3_ and β_6_ (Fig. 4b). Representative images of integrin staining in fibrous, fibrocartilaginous, and cartilaginous regions are depicted in Fig. 6a–c, respectively.

In parallel, for each compartment, the mean vertical score of each integrin was compared (Fig. 4c). Interestingly, in every compartment, the mean expression score for β_6_ integrin was significantly higher than the mean expression score for α_V_β_5_ (Fig. 4c).

The broad and high expression of β_6_ within spinal osteophytes was also emphasized by the correlation between the horizontal score (sum of the compartment scores for one sample) and the total osteophyte size (*r* = 0.474 4, *P* = 0.004) (data not shown). For BMCs and the fibrocartilaginous compartment in particular, the mean α_V_β_5_ expression score was significantly lower than both the α_V_β_3_ and β_6_ scores (Fig. 4c). However, these effects were less significant than those related to the overexpression of β_6_, as small effect sizes were calculated (0.3 for BMCs and 0.3 for the fibrocartilaginous compartment).

Separate graphic representations of the integrin expression scores for osteophytes from the facet joints and degenerative discs compared to those for all samples are presented in Fig. S5.

### Regulation of α_V_, β_5_, β_3_, and β_6_ in OA chondrocytes with fibrosis, inflammation, and mineralization

OA disease involves deep perturbation of the chondrocyte secretory profile.^8^ Notably, the increases in inflammation-, ossification-, and fibrosis-associated markers participate in cartilage matrix degeneration and disease progression.^9,12,35^ To test the regulation of integrins in conditions that increase fibrosis, inflammation, or ossification markers, we used OA primary chondrocytes.

**Fig. 7:**
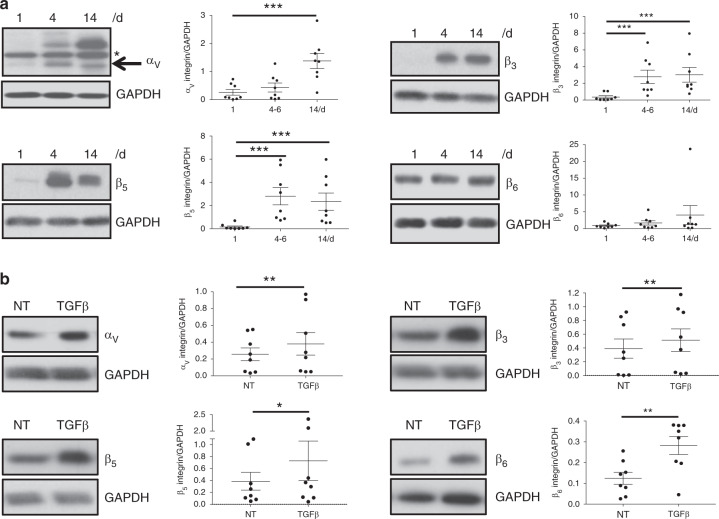
In vitro integrin regulation during chondrocyte dedifferentiation and fibrosis. **a** Freshly isolated human OA chondrocytes were cultivated for 14 days in a monolayer. Cell lysates were performed at the indicated times (days 1, 4, and 14) and subjected to western blotting analysis using specific antibodies directed against α_v_, β_3_, β_5_, and β_6_ integrin subunits. GAPDH detection was used as a loading control, and western blot quantification was performed on several patients (*n* = 8). One-way ANOVA paired test was applied on logarithmic values, and comparisons between time points were performed with Tukey post hoc test. For α_v_, β_3_, and β_5_, *P* < 0.000 1; for β_6_, *P* = 0.272 0. **b** Integrin expression was evaluated by western blotting on NT chondrocytes or treated with TGFβ (10 ng·mL^−1^) for 48 h. A representative image for each integrin subunit is presented. GAPDH detection was used as a loading control, and western blot quantification was performed on several patients (*n* = 8). Comparisons between NT and TGFβ were performed using a nonparametric Wilcoxon paired test. For α_v_, *P* = 0.008; for β_3_ *P* = 0.008; for β_5_ *P* = 0.023; for β_6_ *P* = 0.008

**Fig. 8:**
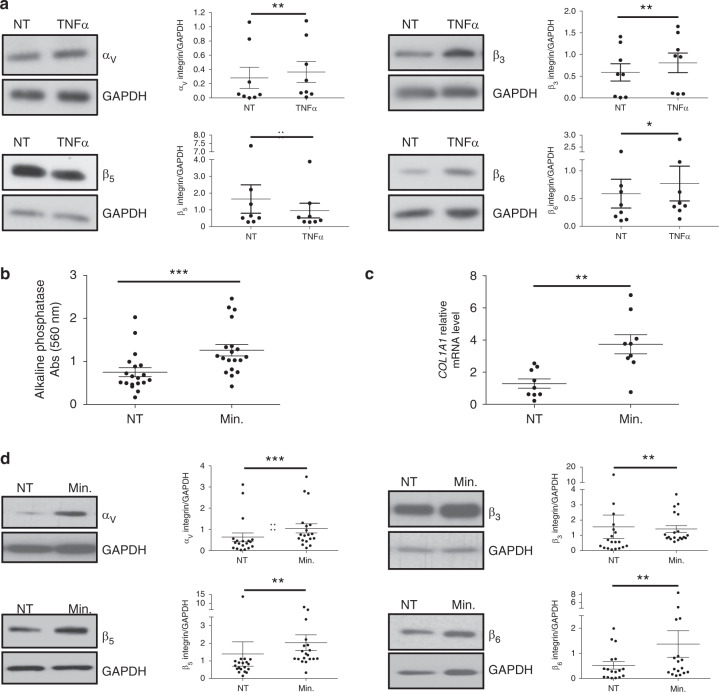
In vitro integrin regulation during chondrocyte inflammation and osteogenic differentiation. **a** The expression of each integrin subunit was evaluated by the western blotting of NT chondrocytes or cells treated with TNFα (10 ng·mL^−1^) for 24 h. Representative western blot images are presented. GAPDH detection was used as a loading control, and western blot quantification was performed on several patients (*n* = 8). Comparisons between NT and TNFα were performed using the nonparametric Wilcoxon paired test. For α_v_, *P* = 0.008; for β_3_ *P* = 0.008; for β_5_ *P* = 0.008; for β_6_ *P* = 0.039. Osteogenic differentiation mix was applied (Min.) or not (NT) to dedifferentiated OA chondrocytes for 3 weeks. Effective mineralization (Min.) was verified by **b** alkaline phosphatase activity measurement (*n* = 19, nonparametric Wilcoxon paired test, *P* < 0.000 1) and **c** RT qPCR analysis of *COL1A1* differential gene expression. Input amounts were normalized to the GAPDH endogenous control gene (*n* = 9, nonparametric Wilcoxon paired test, *P* = 0.009 1). **d** Integrin expression was evaluated within chondrocyte cell lysates by western blotting analyses using specific antibodies. A representative picture of the western blot is presented. GAPDH detection was used as a loading control, and western blot quantification was performed on several patients (*n* = 19). Comparisons between NT and Min were performed using a nonparametric Wilcoxon paired test. For α_v_, *P* = 0.000 2; for β_3_ *P* = 0.007 5; for β_5_ *P* = 0.002 7; for β_6_ *P* = 0.001 8

Round-shaped, freshly isolated chondrocytes cultivated in monolayers undergo spontaneous dedifferentiation, which is notably accompanied by an increase in fibrosis markers, including type III collagen and αSMA,^11^ and show a fibroblastic-like shape.^36^ As depicted in Fig. 7a, the expression of the α_V_, β_3_, and β_5_ integrin subunits was increased during dedifferentiation. Interestingly, the β_6_ subunit was constantly expressed in chondrocytes irrespective of chondrocyte morphology, suggesting a different mode of regulation. TGFβ is a well-known fibrosis inducer that increases the expression of type III collagen and αSMA in OA chondrocytes.^11^ As shown in Fig. 7b, TGFβ increased all integrin subunits studied (α_V_, β_3_, β_5_, and β_6_). Inflammation was studied using stimulation with different cytokines. TNFα treatment increased α_V_, β_3_, and β_6_ expression while decreasing β_5_ expression (Fig. 8a). However, TNFα seemed to have a greater effect on β_3_ and β_6_ than on α_v_ and β_5_ (medium calculated effect size for the latter two integrins). Similarly, IL1β increased β_3_ expression and decreased β_5_ expression but had no effect on α_V_ and β_6_ expression (data not shown). Notably, IL6 had no effect on integrin regulation (data not shown).

In vitro-induced mineralization was achieved by the application of a prototypical osteogenic mix (i.e., dexamethasone, β-glycerophosphate, and ascorbic acid) to chondrocytes for 3 weeks. Mineralization was verified through measurements of alkaline phosphatase (ALP) activity (Fig. 8b) and *COL1A1* mRNA expression (Fig. 8c). As observed by western blotting experiments (Fig. 8d), osteogenic conditions upregulated the expression of all α_V_, β_3_, β_5_, and β_6_ integrin subunits. However, the effect size was large for α_V_ but small for the three other integrin subunits.

In conclusion, we observed in vitro that integrin upregulation parallels increases in fibrosis, inflammation, and ossification markers. Accordingly, integrins may be used to monitor fibrosis, inflammation, or calcification during OA progression.

## Discussion

In this work, we propose a possible relevance for the detection of integrins α_V_β_5_, α_V_β_3_, and α_V_β_6_ by PET/CT using PRGD2 ligands for OA diagnosis. We identified these three integrin complexes as the molecular targets that interacted with the highest affinity with PRGD2 ligands. The α_V_β_5_, α_V_β_3_, and α_V_β_6_ integrins were overexpressed in OA cartilage compared to normal cartilage. Their expression within the different spinal osteophyte compartments was also reported and scored. These three integrins were mainly expressed in osteophytes with fibrous or fibrocartilaginous zones rather than those with pure cartilaginous zones. They were also strongly expressed in blood vessels during the neovascularization of osteophytes as well as in bone lining and BMCs. According to the osteophyte composition and integrin expression, we defined osteophyte compartments/cells that could preferentially take up PRGD2 ligands in vivo. Finally, we observed that integrin subunits in vitro were overexpressed in fibrochondrocytes and chondrocytes undergoing osteogenic differentiation, suggesting that integrin expression might reflect OA-linked processes of fibrosis and mineralization.

In the oncological field, RGD-specific integrin overexpression by tumor cells or neovessels allows integrin-based diagnosis by imaging.^37^ In the rheumatology field, the clinical application of integrin imaging (with a 68Ga-PRGD2 ligand) has been tested (i) in a proof-of-concept study for synovial angiogenesis evaluation in rheumatoid arthritis patients^38^ and (ii) in a retrospective study in which we reported ^18^F-FPRGD_2_ uptake by OA tissues in vivo (i.e., femoral cartilage and spinal osteophytes) in patients undergoing PET/CT for oncologic purposes.^6^ Future prospective studies assessing the clinical relevance of PET/CT in diagnosing OA will be performed with the same PRGD_2_ core used in the retrospective study coupled to another positron emitter, ^68^Ga. In this work, we showed that the bioconjugation of Ga chelators (NOTA or NODAGA) to PRGD_2_ has minimal effect on integrin selectivity and affinity. This result would tip the balance toward the selection of NODAGA-PRGD_2_ for future clinical studies, as NODAGA presents better chelator properties for ^68^Ga as well as a better bioconjugation rate.^32^

This work confirms integrin α_V_β_3_ as a specific target of the core PRGD_2_ ligand.^23^ Moreover, we identified integrin α_V_β_5_ and α_V_β_6_ as additional targets with high affinity for the PRGD_2_ ligand, whereas α_5_β_1_ was the complex with the lowest affinity (IC_50_ ≈ 600 nmol·L^−1^). In contrast, competition studies performed with immobilized integrins showed that RGD_2_ recognized α_5_β_1_ with nanomolar affinity, similar to α_V_β_3_ and α_V_β_5_.^39^ This modified ligand selectivity could be explained by the attachment of a PEG (polyethylene glycol) moiety to the core RGD_2_ (termed PRGD_2_). The IC_50_ measured for α_5_β_1_ suggests that this complex would not be targeted in vivo by the ligand. Furthermore, we observed no difference in α_5_β_1_ expression between normal and OA cartilage (Fig. S2), suggesting that α_5_β_1_ might not be useful to distinguish OA progression.

By contrast, we showed in situ that the α_V_β_5_ and α_V_β_3_ complexes and the β_6_ subunit are significantly increased in hip OA cartilage compared to normal cartilage. RGD-specific integrin expression in normal cartilage is consistent with the physiological role of integrin. Integrins from the β_1_, β_3_, and β_5_ families were reported to mediate the interaction of chondrocytes with matrix proteins^25,40^ and the attachment to cartilage^24^ as well as to serve as natural mechanosensors.^26^ Accordingly, α_V_β_3_, α_V_β_5_, and α_5_β_1_ heterodimer expression was detected by IHC in human normal knee cartilage sections.^29,30^ Non-RGD integrin subunits (namely, α_2_, α_4_, and β_2_ integrin) were reported to be increased in OA human femoral cartilage compared to normal cartilage,^27,28,30,41^ whereas RGD-specific integrin overexpression in OA cartilage has rarely been reported.

In this work, we also characterized for the first time the histological composition of spinal osteophytes (from facet joints and degenerative discs) and reported and scored α_V_β_5_, α_V_β_3_, and β_6_ integrin expression within the whole osteophyte surface and each histological compartment. We first transposed the standardized histological classification described for knee osteophytes^34^ for use in spinal osteophytes. Among the 35 samples from 9 patients who were analyzed, we found a majority of type C (60%) and D (25.7%) osteophytes. Similarly, Junker et al. analyzed 97 knee samples from 10 patients and found a majority of type D osteophytes, corresponding to the most advanced osteophyte stage.^34^ This could be explained by the advanced stage of the disease requiring chirurgical ablation and by the patient age (mean = 64.6; range 38–88). In addition to this established classification, we observed overlapping expression of type II and III collagen protein in situ, which defined fibrocartilaginous areas within 94.3% of spinal osteophytes. Such areas were previously observed within knee osteophytes at the RNA level through in situ hybridization experiments.^14^ Similar to knee osteophytes,^42^ blood vessels could be found in 94.3% of samples mainly within the bone area of the analyzed spinal osteophytes.

Interestingly, ^18^FPRGD_2_ uptake was not detected in all osteophytes present in the vertebral columns of patients.^6^ Given the selectivity of PRGD_2_ for α_v_β_5_, α_v_β_3_, and α_v_β_6_, it might be expected that osteophytes expressing the highest amounts of these integrins would be targeted by radiolabeled PRGD_2_.

We found that integrin expression in osteophytes was present in both the early (types A and B) and late stages (types C and D) and showed upregulation during disease progression. This emphasizes the relevance of a diagnostic method highlighting integrin expression, which would therefore allow early diagnosis and follow-up of disease progression. Moreover, we found that the highest mean expression scores for α_v_β_5_, α_v_β_3_, and α_v_β_6_ were attributed to blood vessels, cells from the bone lining and fibrous areas. In comparison, the lowest mean α_v_β_5_ expression scores were found for osteocytes and cartilaginous areas. Taken together, these results suggest that osteophytes predominantly containing fibrous areas and/or bone areas with a high ratio (cells from the bone lining/osteocytes) and blood vessels would preferentially show radiolabeled PRGD_2_ uptake.

The α_V_β_3_ and α_V_β_5_ staining observed in vessels (in the endothelial wall) from spinal osteophytes indicates new vessel formation (angiogenesis). Indeed, integrins are implicated in tumor cell growth, invasion, and metastasis as well as angiogenesis. α_V_β_3_ and α_V_β_5_, in particular, are known to be highly expressed by activated endothelial cells in the tumor neovasculature but weakly expressed in resting endothelial cells and in most normal tissues and organs.^43^ Notably, the observation of β_6_ expression within endothelial cells is in line with data reporting α_V_β_6_ expression in the epithelium from other tissues, such as lung, kidney, skin, enamel, and intestine.^44^ Interestingly, integrin staining was also noticed on the external fibrous part of the blood vessels (i.e., “adventice”), which is consistent with the high mean expression score for integrin associated with this compartment. Indeed, all PRGD_2_-selective integrins show high mean expression scores in fibrous areas.

We also provided in situ evidence of strong integrin α_V_β_3_, α_V_β_5_, and β_6_ expression within cells from the bone lining, which is in contrast with the low scores for integrin expression in osteocytes present in spinal osteophytes. Similar heterogeneous expression of α_V_ integrin by osteocytes compared to that in osteoblasts was previously reported.^45^ However, most studies reporting integrin expression have mainly been performed with bone cell cultures (i.e., primary human osteoblasts (HOBs), primary human osteoprogenitor cells, primary rat osteoblasts, and mouse MC3T3H1 osteoblasts).^45,46^ For example, the α_V_β_1_, α_V_β_3_, α_V_β_5_, β_6_, and α_V_β_8_ integrins were visualized by immunocytofluorescence at focal adhesion sites of cultured HOBs.^47^ α_5_β_1_, α_V_β_5_, and α_V_β_3_ were found to be constitutively expressed on the cell surface of normal human bone cells by flow cytometric analysis.^48^ Only a few studies have reported the in situ expression of integrins in bone cells, such as β_1_ in osteoblasts and osteocytes from human femoral head osteophytes.^49^ Interestingly, a punctate pattern of β_3_ staining was described in situ along the canalicular wall of osteocytes.^50^ Similarly, we also noticed such punctate patterns for the integrin staining of spinal osteophyte samples (Fig. S6).

In all compartments, β_6_ was the most highly expressed integrin compared to α_V_β_3_ and α_V_β_5_. The integrin expression observed in this work contrasted with the results obtained in fibrocartilaginous cells of porcine vertebral discs (i.e., anulus fibrosus cells), which showed moderate to high β_3_ and β_5_ subunit staining and no β_6_ expression.^51^

Finally, studies have also suggested that RGD integrin overexpression might reflect OA progression. Similarly, increased expression of the α_5_^52^ and α_V_^53^ integrins was associated with OA severity in a meniscectomy model in rodents.

In this work, we observed that integrin expression is increased in chondrocytes with fibrosis and calcification, similar to that encountered in OA cartilage.

We showed that the α_V_, β_3_, and β_5_ integrin subunits were upregulated in fibrochondrocytes compared to freshly isolated chondrocytes. These results confirmed the increased staining of α_V_β_3_ observed after 2 weeks of culture that we^6^ and other researchers^54^ previously reported. In addition, we observed that 14 days of monolayer culture is sufficient to increase α_V_β_5_ surface expression in OA chondrocytes, which has already been described after five passages of chondrocytes.^29^ In line with this, blocking RGD integrin with echistatin^55^ or specifically with α_v_β_5_ antibody^56^ prevented chondrocyte dedifferentiation. In particular, α_V_β_5_ was described as a potent regulator of chondrogenic loss by promoting *COL2* and *ACAN* gene downregulation. In contrast to other subunits, β_6_ was detected in freshly isolated chondrocytes. This might reflect the higher level of β6 detected in situ (44.8%) compared to the levels of α_V_β_3_ (23.7%) and α_V_β_5_ (19.9%) in normal hip cartilage. However, β_6_, as well as α_V_, β_3_, and β_5_, were upregulated by TGFβ, meaning that fibrosis could regulate these four integrin subunits during OA development.

In addition to fibrosis, inflammation seems to play an important role in integrin regulation. Interestingly, TNFα increased α_V_, β_3_, and β_6_ expression but decreased β_5_ expression. α_V_β_3_ is also upregulated by TNFα in human chondrosarcoma cells.^57^ In macrophages, the heterodimer α_V_β_5_ is upregulated by IL4 and PPARγ agonist stimulation.^58^ Moreover, TNFα and IL1β induce the production of the α1 integrin subunit in human skin fibroblasts and MG-63 cells. Interestingly, TGFβ potentializes the effect of IL1β.^59^

Our data also indicate that the osteogenic differentiation of chondrocytes enhanced the upregulation of the single integrin subunit α_V_ and, to a lesser extent, β_3_, β_5_, and β_6_. Previous studies reported that integrin α_5_ expression is increased during the osteogenic differentiation of mesenchymal stem cells.^60^ Other bone-inductive stimuli, such as BMP-2, also prompted increases in integrin (i.e., α_v_β_3_, α_v_β_5_, and α_v_β_6_) expression in HOBs.^47^ Furthermore, the parallel increases in β_6_ protein and *COL1A1* gene expression we observed in chondrocytes were also observed in epithelial cells, where procollagen type I and β_6_ were colocalized, as observed by immunofluorescence.^61^ In contrast, the loss of α_V_β_6_ caused hypomineralization in humans^62^ and mice,^63^ suggesting an association between integrin expression and mineralization.

In conclusion, in this work, we propose that the integrins α_V_β_5_, α_V_β_3_, and α_V_β_6_ might be potential targets for the functional imaging of OA by PET/CT using PRGD_2_-based ligands. Moreover, for the first time, we report differential α_V_β_5_, α_V_β_3_, and β_6_ expression in OA-associated structures (femoral cartilage and spinal osteophytes) previously reported to show PRGD_2_ uptake in vivo. Finally, we propose that integrin overexpression might be used as a diagnostic tool to visualize fibrosis, inflammation and mineralization to potentially monitor OA progression. Further PET/CT experiments using ^68^Ga-NODAGA-PRGD_2_-based ligands should be conducted in a clinical study including OA patients to confirm the prognostic clinical relevance of the α_V_β_5_, α_V_β_3_, and α_V_β_6_ integrins for OA diagnosis.

## Materials and methods

### Competition experiments

The following buffers were prepared: integrin binding buffer (IBB), 20 mmol·L^−1^ Tris pH 7.4, 150 mmol·L^−1^ NaCl, 2 mmol·L^−1^ CaCl_2_, 1 mmol·L^−1^ MgCl_2_, and 1 mmol·L^−1^ MnCl_2_; sample and washing buffer, IBB supplemented with 0.5% bovine serum albumin (BSA); and blocking solution, IBB supplemented with 2% BSA. The five recombinant human (rh) integrins were obtained from R&D Systems: rh integrin α_5_β_1_ (ref: 3230-A5), rh integrin α_v_β_1_ (ref: 6579-AV), rh integrin α_v_β_3_ (ref: 3050-AV), rh integrin α_v_β_5_ (ref: 2528-AV), and rh integrin α_v_β_6_ (ref: 3817-AV). For each integrin complex, a 1.8 µg·mL^−1^ solution was prepared in IBB. Echistatin was radioiodinated by the conjugation of a radioiodinated benzoate (^125^I-SIB) to the epsilon amino group of the lysine side chains. Nunc Maxisorp Module plates were coated overnight at 4 °C with 100 µL of integrin complex at 1.8 µg·mL^−1^. After washing, the plate was incubated with blocking solution. ^125^I-SIB-labeled echistatin was mixed with sample buffer (total binding: control well) or with variable concentrations of ligand. Three ligands were tested (PRGD2, Ga-NOTA-PRGD2 and Ga-NODAGA-PRGD2); unlabeled echistatin was used as a positive control; and NOTA and NODAGA alone were used as negative controls. Then, the prepared solutions were added to the plates, which were incubated for 2 h at room temperature with agitation. After three rinsing steps, the solutions were removed from the plates, and the radioactivity due to binding was counted using a gamma counter. The percentage of the relative binding of radiolabeled echistatin was calculated as (radioactivity in test well/radioactivity in reference well) × (total binding without ligand in the reference well).

### Subject recruitment

Tissue collection was performed in collaboration with the orthopedic surgery and neurosurgery departments (CHU Sart-Tilman, ULg). Human OA cartilage tissue was obtained from patients undergoing hip replacement surgery. Thirty-five spinal osteophytes were obtained after lumbar canal laminectomy or cervical arthrodesis from patients undergoing surgery. Among these 35 osteophytes, 23 were obtained from OA facet joints, and 12 were obtained from degenerative discs (Table S1). OA cartilage [*n* = 37, 24 females, 13 males, mean age = 68.7 years (range 47–86), mean BMI = 30.25 kg·m^−2^ (range 16.4–38.9)] was used for dedifferentiation (*n* = 8), TNFα and TGFβ stimulation (*n* = 8), and osteogenic differentiation (*n* = 19) experiments. Immunohistological staining was performed on normal articular cartilage sections obtained from subcapital femoral neck fractures [*n* = 4, 3 females, 1 male, mean age = 80.8 years (range 78–85), mean BMI = 22.1 kg·m^−2^ (range 18.9–25.7)], OA cartilage [*n* = 8, 7 females, 1 male, mean age = 54.6 years (range 23–84), mean BMI = 23.9 kg·m^−2^ (range 18.2–31.2)] and spinal osteophytes [*n* = 9, 5 females and 4 males, mean age = 64.6 years (range 38–88), mean BMI = 28.4 kg·m^−2^ (range 21.1–41.0)]. Informed consent from each patient was obtained and approved by the Research Ethics Committee of CHU de Liège, Belgium, as previously described.^64^

### PET/CT image acquisition and analysis

PET/CT image acquisition and analysis were performed as previously described for patients suffering from locally advanced rectal adenocarcinoma or renal masses in whom the musculoskeletal uptake of ^18^F-FPRGD_2_ was observed in locations corresponding to hip OA, and peridiscal lumbar osteophytes were performed as previously described.^6^

### IHC and staining experiments

IHC analyses were performed on sections of human femoral cartilage and spinal osteophytes. Upon reception, samples were fixed in 4% paraformaldehyde, decalcified in DC2 (VWR international, Fontenay-sous-Bois, France), and embedded in paraffin. Immunohistochemistry was performed on tissue sections (5 μm) after dewaxing and chondroitinase unmasking using specific anti-antibodies [α_V_β_5_ (MAB2019Z), α_V_β_3_ (MAB1976), Merck-Millipore, Darmstadt, Germany; β_6_ (AB233519), type III collagen (AB6310), Abcam, Cambridge, Massachusetts, USA and type II collagen (sc-52658), Santa-Cruz Technologies, Dallas, Texas, USA]. Rinsed sections were incubated with Envision+ System-HRP Labeled Polymer anti-rabbit (#K4003, Agilent, Santa Clara, California, USA). Peroxidase was detected with a Liquid DAB + Substrate Chromogen System (#K3468, Agilent, Santa Clara, California, USA). Rinsed sections were counterstained with Carazzy’s hematoxylin. Sections incubated without primary antibody served as negative controls. Histological staining with hematoxylin/eosin, alizarin red and Masson’s trichrome was performed according to standard protocols.

### Cell culture

Human OA chondrocytes were isolated from human cartilage as previously described.^11^ For the dedifferentiation experiment, freshly isolated chondrocytes were cultured in a monolayer for 14 days in DMEM (Lonza, Basel, Switzerland), 10% FCS (Lonza, Basel, Switzerland), l-glutamine (2 mmol·L^−1^), streptomycin (100 mg·mL^−1^), and penicillin (100 U·mL^−1^) (BioWhittaker, Walkersville, Maryland, USA). Dedifferentiated chondrocytes were treated with 10 ng·mL^−1^ TGFβ (Sigma-Aldrich, Saint Louis, Missouri, USA) for 48 h and with 10 ng·mL^−1^ TNFα (Biosource, San Diego, California, USA) for 24 h.

### Osteogenic differentiation and ALP activity measurement

Osteogenic differentiation was achieved by incubating OA dedifferentiated chondrocytes with DMEM supplemented with 10 mmol·L^−1^ β-glycerophosphate, 60 mmol·L^−1^ ascorbic acid, and 10^−7^ mol·L^−1^ dexamethasone for 3 weeks. The medium was replaced every 3–4 days. ALP expression was detected after 3 weeks. Briefly, plated cells were fixed with 70% (v/v) ethanol and then stained with 0.3 mg·mL^−1^ naphthol AS-MX phosphate (#N9252, Sigma-Aldrich, St. Louis, Missouri, USA), 0.005 mg·mL^−1^ NN dimethylformamide diluted in Tris buffer (0.2 mol·L^−1^, pH 9.1) and 1 mg·mL^−1^ Fast Blue Salt BB (#F3378, Sigma-Aldrich) added extemporaneously. Cells were then incubated with a solution of 0.05 N NaOH diluted in ethanol before the absorbance was detected at 560 nm.

### Western blotting

Whole cell lysates were separated by sodium dodecyl sulfate–polyacrylamide gel electrophoresis as explained earlier.^11^ Membranes were incubated with the following primary antibodies: from Cell Signaling Technology, Danvers, Massachusetts, USA, anti-α_V_ (#4711 S), -β_3_ (#13166 S) and -β_5_ (#3629 S) integrins; from Abcam, Cambridge, Massachusetts, USA, anti-β_6_ integrin (#187155); and from Sigma-Aldrich, St. Louis, Missouri, USA, anti-GAPDH (#G9545). Anti-rabbit secondary antibodies (Cell Signaling) and ECL chemiluminescent reagents (Amersham Biosciences, Diegem, Belgium) were used for revelation.

### Real-time qPCR

Total RNA was extracted from dedifferentiated chondrocytes left untreated or incubated with osteogenic differentiation mix using a Nucleospin RNA Extraction Kit (#740955, Macherey-Nagel, Düren, Germany). cDNA was next synthesized by reverse transcription with a RevertAid H Minus First Strand cDNA Synthesis Kit (#K1632, Thermo Scientific, Pittsburgh, Pennsylvania, USA) and amplified by PCR using a KAPA SYBR FAST detection system (#KK4611, Sopachem, Eke, Belgium). Real-time RT-PCR experiments were run on a LightCycler 480 instrument (Roche Diagnostics GmbH, Mannheim, Germany), and data were analyzed using LC480 software release 1.5.0 SP4. The 2^−ΔΔCt^ method was used to calculate the relative gene expression between untreated chondrocytes and chondrocytes undergoing osteogenic differentiation. Input amounts were normalized to the GAPDH endogenous control gene. All primers were purchased from Eurogentec, Seraing, Belgium.

### Data analysis

For competition experiments, the radioactivity of ^125^I-SIB-echistatin bound to the cells in the absence of ligand was determined and expressed as the mean cpm value (used as the reference). For each ligand concentration, the relative binding of ^125^I-SIB-echistatin was then calculated, and the values were plotted against the logarithm of the ligand concentration. The experimental data were subjected to nonlinear regression using a five-parameter logistical model with GraphPad Prism 5 software. The IC_50_ values were determined by GraphPad Prism software. For cartilage IHC images, the number of DAB-positive cells was calculated blindly using Cytomine software.^65^ The normal and OA groups were compared using the nonparametric Mann–Whitney *U* test. For ALP activity measurements, the absorbance values were directly plotted on the graph. For western blotting quantification, Image Studio Lite software was used to determine the intensity of each band. The values reported on graphs correspond to the ratio of the intensities (protein/GAPDH) for each patient analyzed. Comparisons among three groups were performed using one-way ANOVA with normalized values. Comparisons between two groups were performed using the Wilcoxon matched-pairs signed rank test. Comparisons among mean integrin expression scores from different osteophyte compartments were performed with one-way ANOVA and the Kruskal–Wallis test. *P* values were considered significant when they were <0.05. Sample size power analyses were performed for all experiments using the Cohen coefficient calculation (κ) method, which defines the confidence level (or effect size) as follows: very small (0.01);^66^ small (0.2);^67^ medium (0.5);^67^ large (0.8);^67^ very large (1.2)^66^, and huge (2);^66^ the last value represents the optimal coefficient. All calculated effect sizes were close to or greater than large unless stated otherwise in the text.

## Supplementary information

Figure S1

Figure S2

Figure S3

Figure S4

Figure S5

Figure S6

Table S1

Table S2

Table S3

Table S4
