# Supplementary material for: Toward diagnostic relevance of the αVβ5, αVβ3, and αVβ6 integrins in OA: expression within human cartilage and spinal osteophytes
Source: Bone Res. 2020 Sep 30;8:35. doi: 10.1038/s41413-020-00110-4 (PMC7527564; doi:10.1038/s41413-020-00110-4)
Supplement: Supplementary file 1 — Figure S1 [file 41413_2020_110_MOESM1_ESM.pdf]

Figure S1

$PRGD_2$

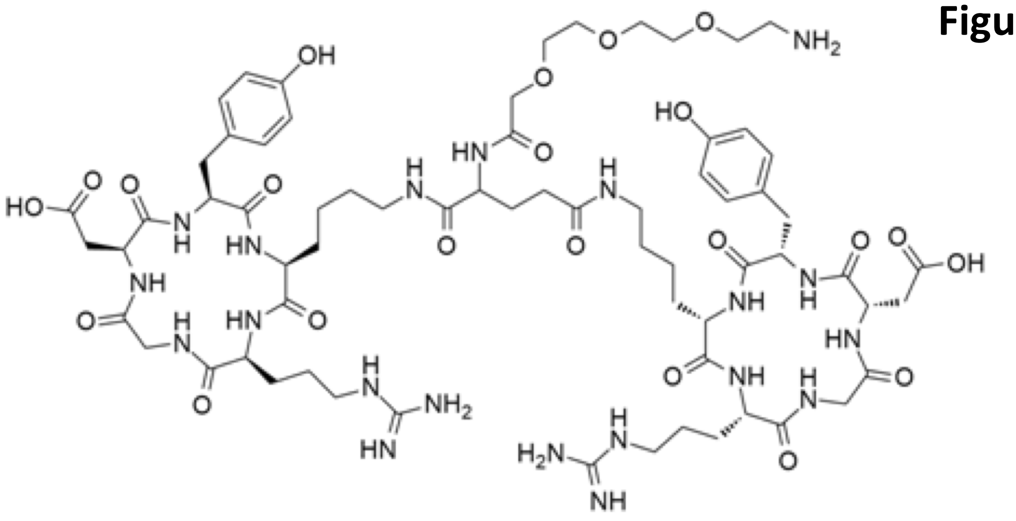

NOTA-$PRGD_2$

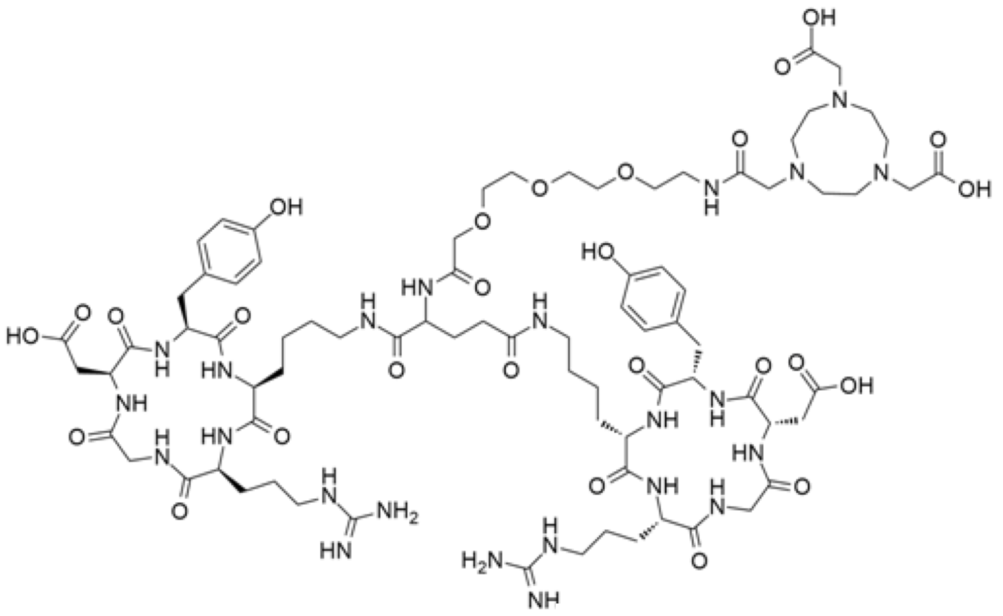

NODAGA-$PRGD_2$

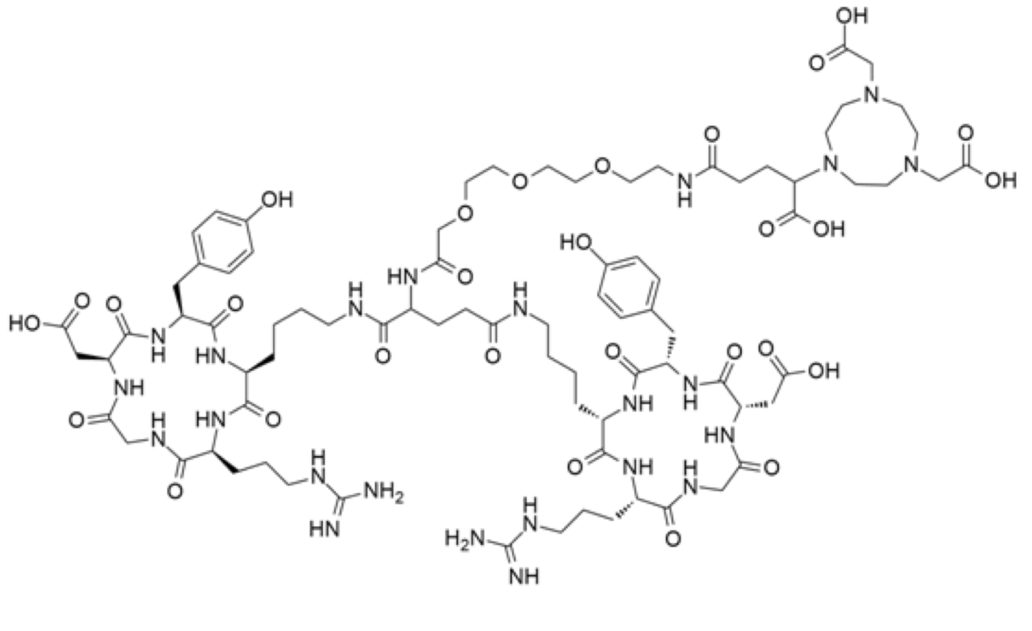

Figure S1. Chemical structures of $PRGD_2$ ligands
Adapted from Salvé *et al.*, 2018 [32]
