# Supplementary material for: Toward diagnostic relevance of the αVβ5, αVβ3, and αVβ6 integrins in OA: expression within human cartilage and spinal osteophytes
Source: Bone Res. 2020 Sep 30;8:35. doi: 10.1038/s41413-020-00110-4 (PMC7527564; doi:10.1038/s41413-020-00110-4)
Supplement: Supplementary file 2 — Figure S2 [file 41413_2020_110_MOESM2_ESM.pdf]

a. Integrins expression along cartilage zone

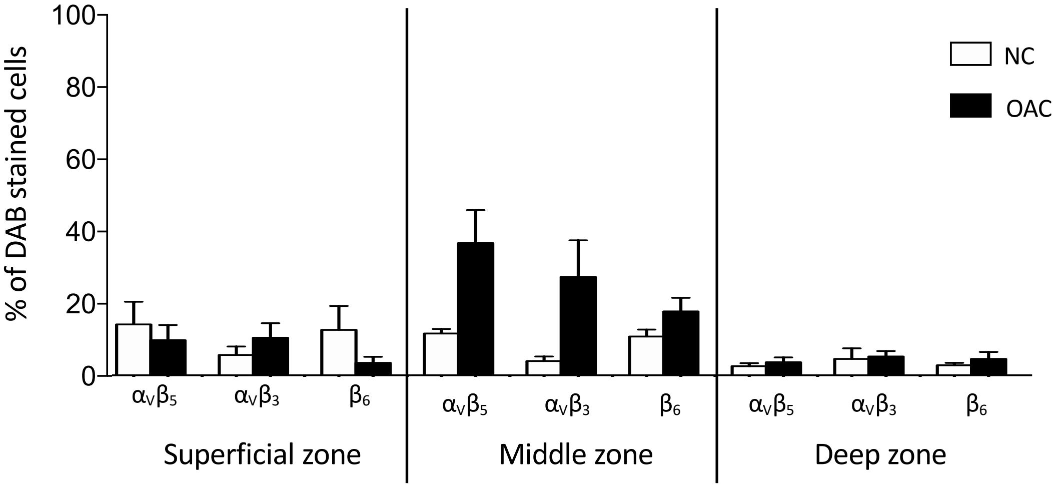

b. α5β1 integrin staining

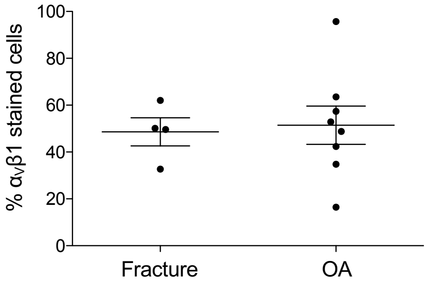

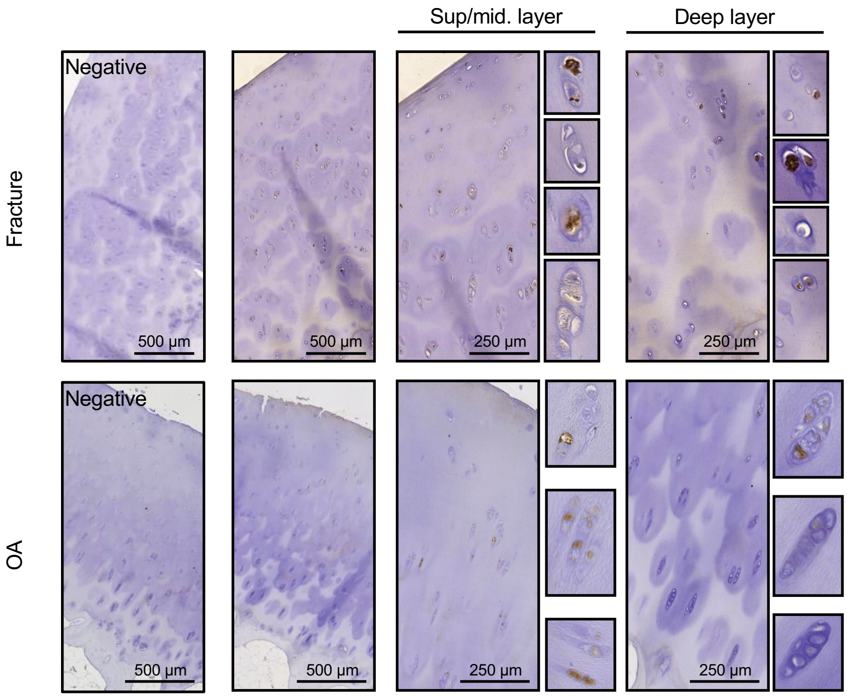

Figure S2. (a) Graphical representation of integrins expression along cartilage zone. The percentage of stained cells using $\alpha_V\beta_5$, $\alpha_V\beta_3$ and β_6 specific antibodies in normal cartilage (NC, white) and OA cartilage (OAC, black) is presented for superficial, middle and deep zones. **(b) Integrin $\alpha_5\beta_1$ expression within healthy (fracture) and OA human cartilage.** Representative IHC pictures of human cartilage sections from hip fracture (upper panel) or OA hip (down panel) stained with anti-$\alpha_5\beta_1$ antibody. Analysis was done on several patients and the percentage of positively stained cells was not significantly different in OA hip (n=8) compared to hip fracture (n=4) (Mann Whitney test for comparisons: p=0.9333). Scale bars are reported on each picture.
