# Supplementary material for: Toward diagnostic relevance of the αVβ5, αVβ3, and αVβ6 integrins in OA: expression within human cartilage and spinal osteophytes
Source: Bone Res. 2020 Sep 30;8:35. doi: 10.1038/s41413-020-00110-4 (PMC7527564; doi:10.1038/s41413-020-00110-4)
Supplement: Supplementary file 3 — Figure S3 [file 41413_2020_110_MOESM3_ESM.pdf]

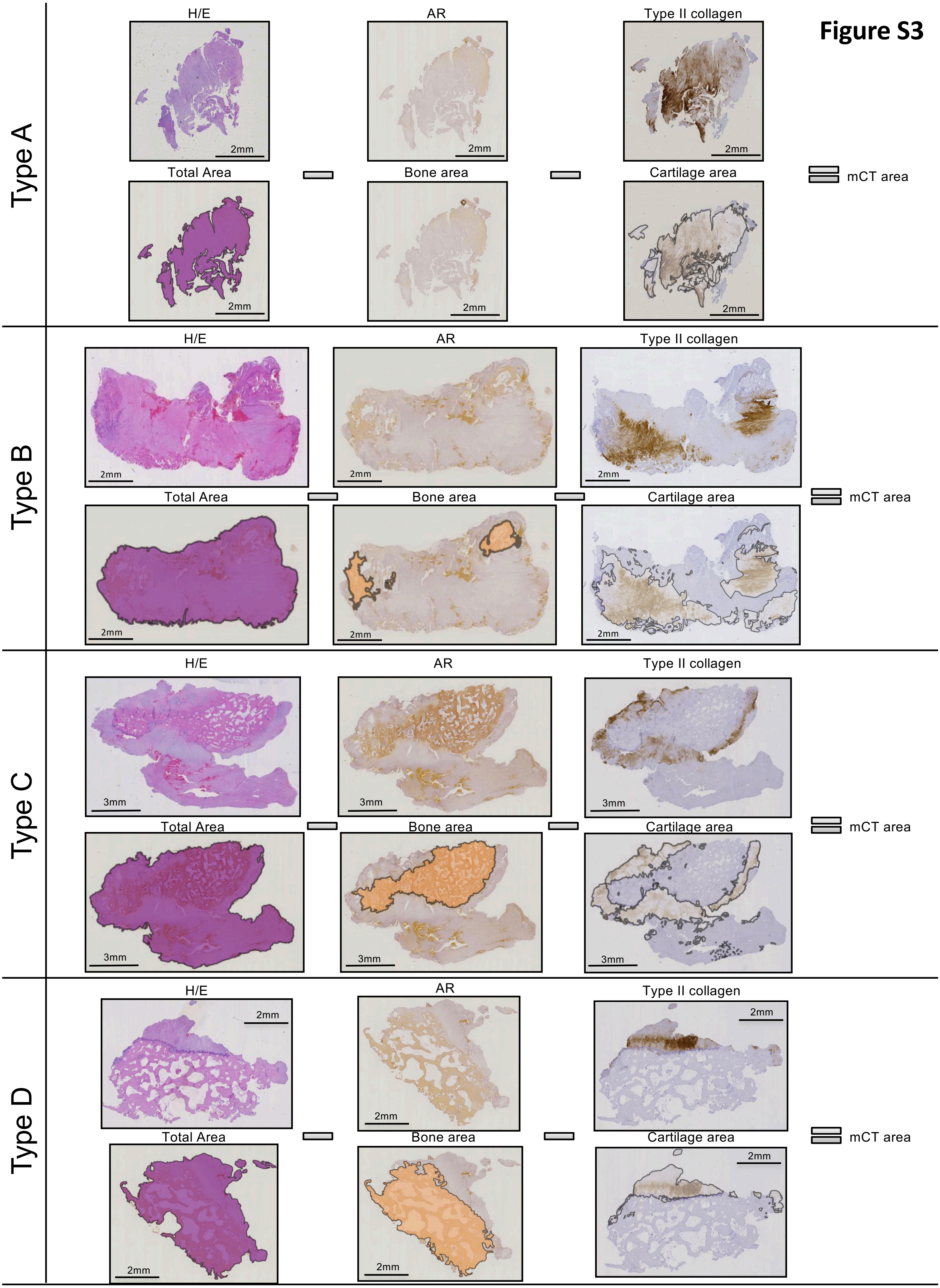

Figure S3. Human spinal osteophytes histological classification A representative picture of each osteophyte type is presented. According to Junker's classification [36], proportion of bone, cartilage and mesenchymal connective tissue (mCT), defines 4 osteophytes types: A, B, C or D. For each osteophyte type, (immuno) stainings are presented on the upper panels, and lower panels show total (violet), bone (orange) and cartilage (white) areas, delimited and calculated from the upper stainings with Cytomine software. The substraction of bone and cartilage areas from the total area allow the determination of mCT area within osteophytes samples. 35 human spinal osteophytes samples (from 9 patients) were subjected to hematoxylin/eosine (H/E), alizarin red (AR) stainings and type II collagen immunostaining. Complementary, all areas values (mm^2) and percentage of bone and mCT (useful for osteophyte type determination) for each osteophyte sample are indicated in **Table 1.**
