# Supplementary material for: Toward diagnostic relevance of the αVβ5, αVβ3, and αVβ6 integrins in OA: expression within human cartilage and spinal osteophytes
Source: Bone Res. 2020 Sep 30;8:35. doi: 10.1038/s41413-020-00110-4 (PMC7527564; doi:10.1038/s41413-020-00110-4)
Supplement: Supplementary file 4 — Figure S4 [file 41413_2020_110_MOESM4_ESM.pdf]

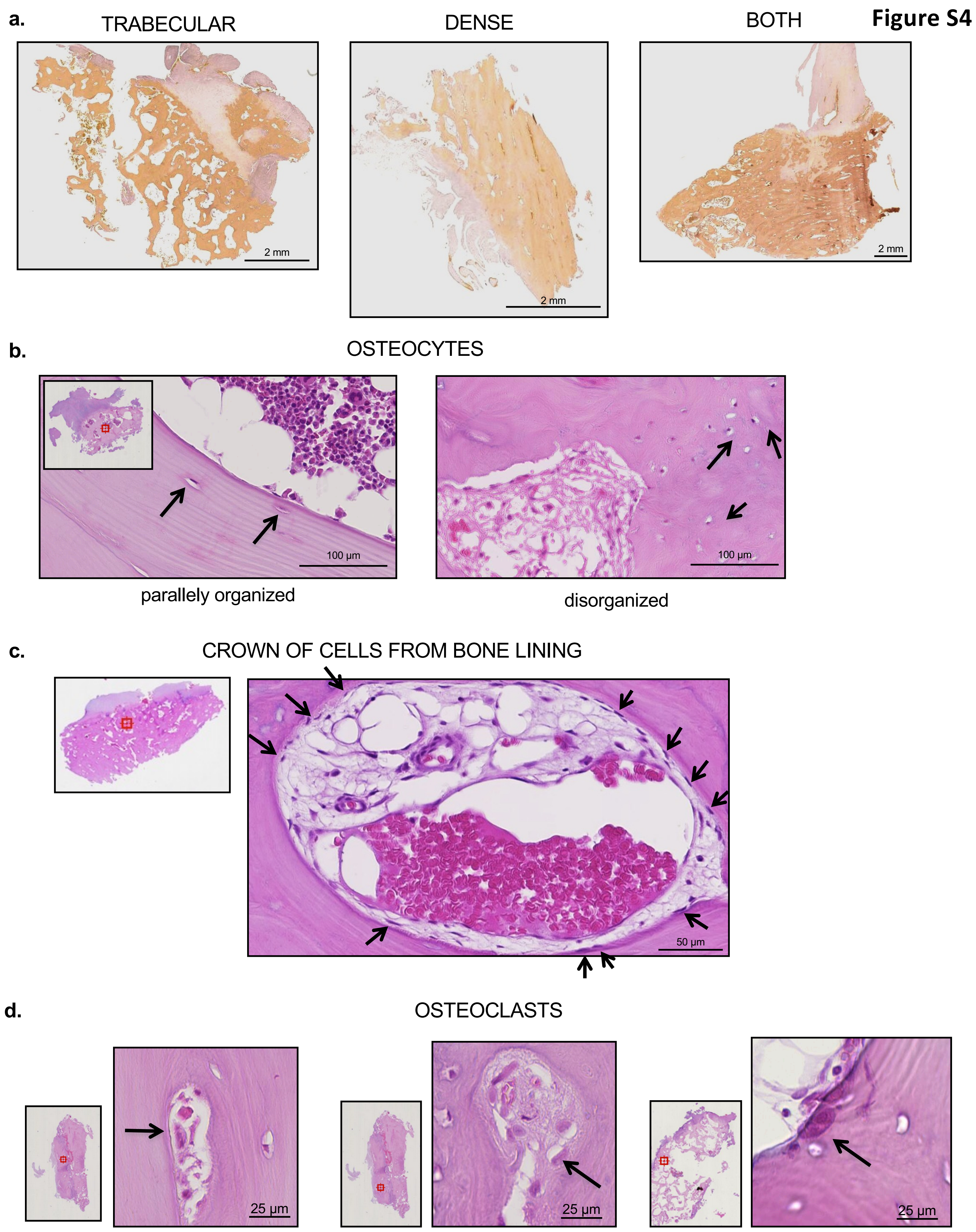

Figure S4. Human spinal osteopyte bone compartment description

(a) Representatives pictures of alizarin red staining showing bone types encountered within osteophytes samples are presented. **(b)** Representative picture of hematoxyline/eosine staining showing osteocytes distributed in bone matrix presenting a lamellar parallel organization or in woven bone matrix organization, **(c)** cells from the bone lining organized in crown and **(d)** remodelling bone zones, where osteoclasts are indicated with a black arrow. Scale bars are reported on each picture.
