# Supplementary material for: Toward diagnostic relevance of the αVβ5, αVβ3, and αVβ6 integrins in OA: expression within human cartilage and spinal osteophytes
Source: Bone Res. 2020 Sep 30;8:35. doi: 10.1038/s41413-020-00110-4 (PMC7527564; doi:10.1038/s41413-020-00110-4)
Supplement: Supplementary file 5 — Figure S5 [file 41413_2020_110_MOESM5_ESM.pdf]

Figure S5

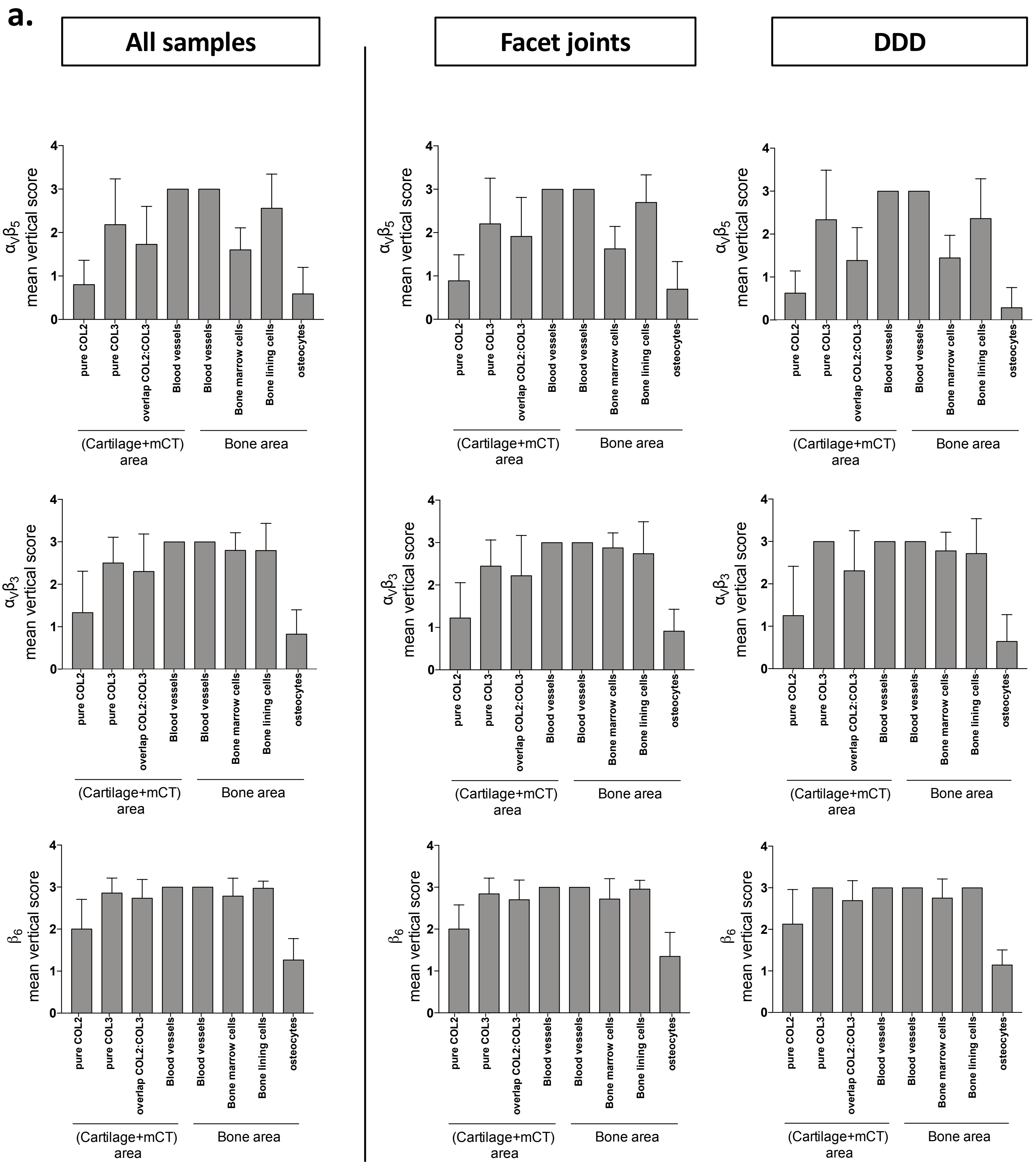

a.
All samples
Facet joints
DDD
$\alpha_v\beta_5$ mean vertical score
$\alpha_v\beta_3$ mean vertical score
β_6 mean vertical score
0
1
2
3
4
pure COL2
pure COL3
overlap COL2:COL3
Blood vessels
Blood vessels
Bone marrow cells
Bone lining cells
osteocytes
(Cartilage+mCT) area
Bone area

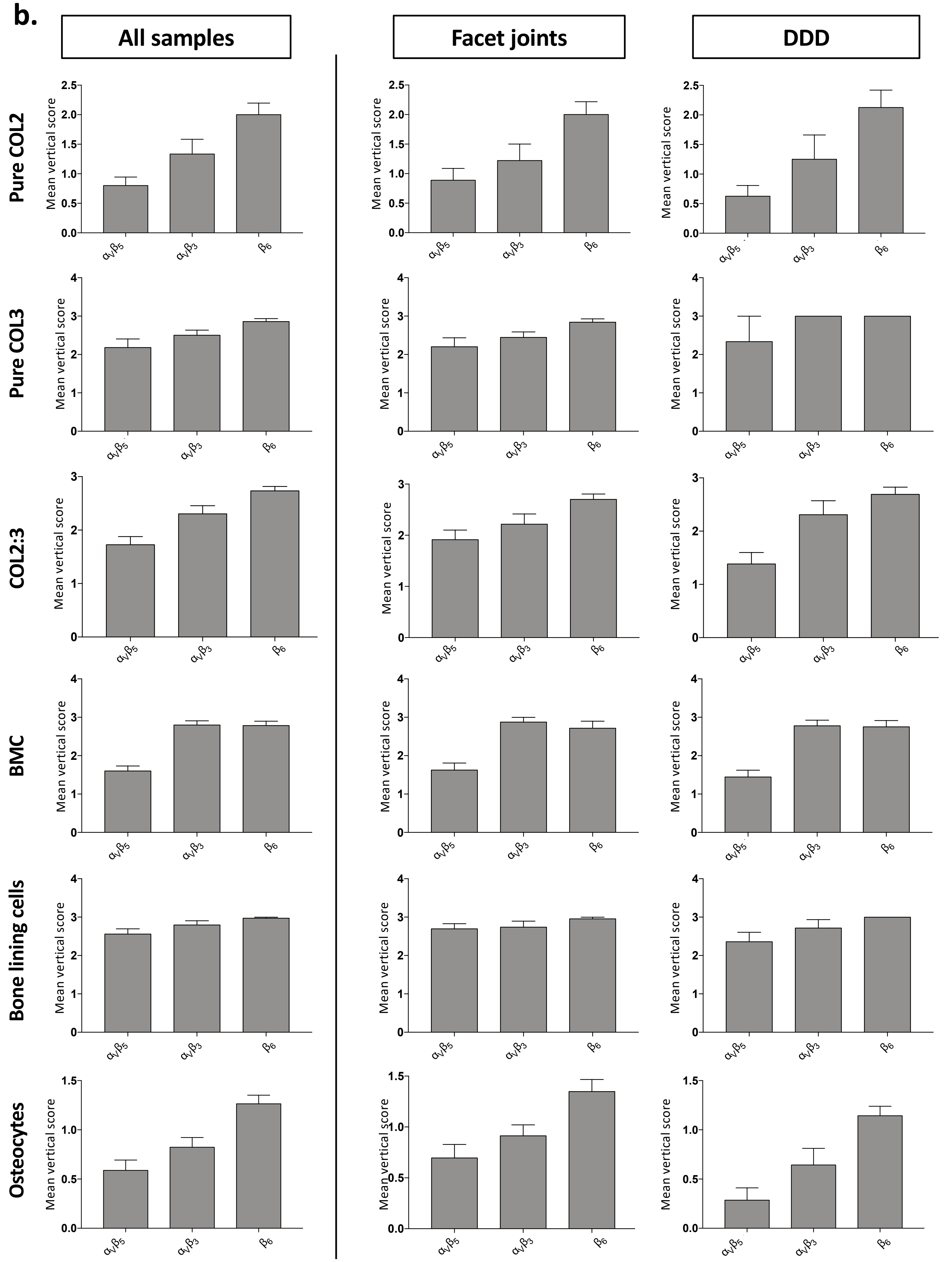

Figure S5. Graphic representation of integrin expression scores within spinal osteophytes. For each integrin the mean vertical expression scores were calculated for each compartment. **(a)** For each integrin staining, the mean vertical expression scores obtained for each compartment/cell type were compared for all osteophytes (n=35), for osteophytes from FJ (n=23) and for osteophytes from DDD (n=12). **(b)** For each compartment/cell type, the mean vertical expression score of each integrin was compared for all osteophytes (n=35), for osteophytes from FJ (n=23) and for osteophytes from DDD (n=12).
