# Supplementary material for: Toward diagnostic relevance of the αVβ5, αVβ3, and αVβ6 integrins in OA: expression within human cartilage and spinal osteophytes
Source: Bone Res. 2020 Sep 30;8:35. doi: 10.1038/s41413-020-00110-4 (PMC7527564; doi:10.1038/s41413-020-00110-4)
Supplement: Supplementary file 6 — Figure S6 [file 41413_2020_110_MOESM6_ESM.pdf]

Figure S6

a. anti-$\alpha_V\beta_5$

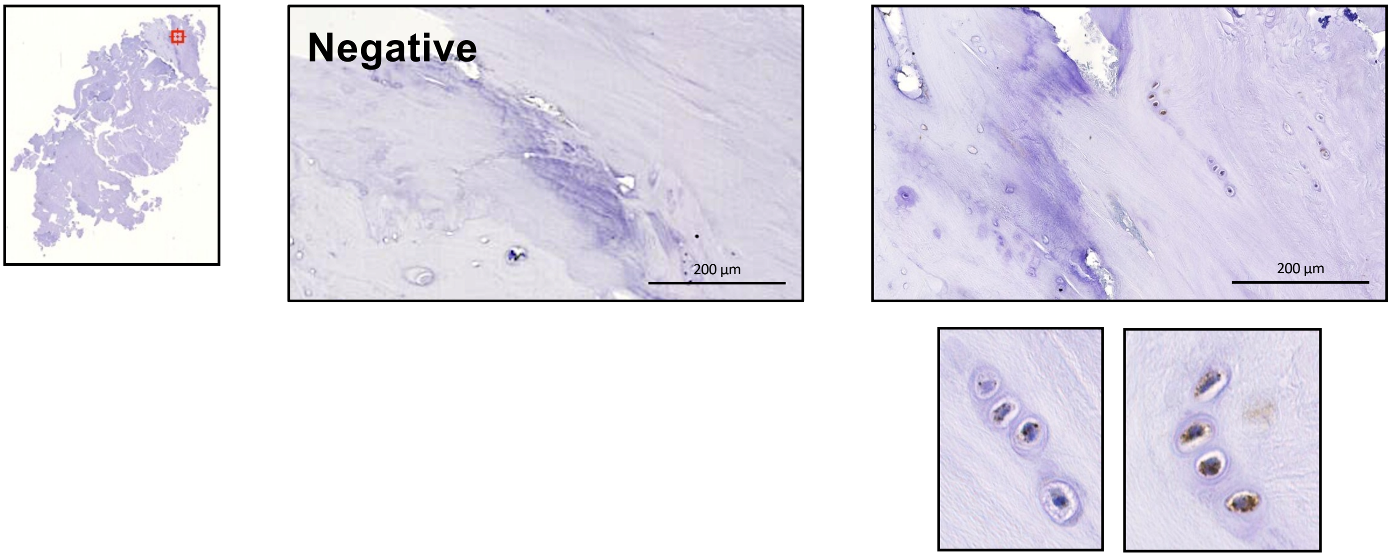

b. anti-$\alpha_V\beta_3$

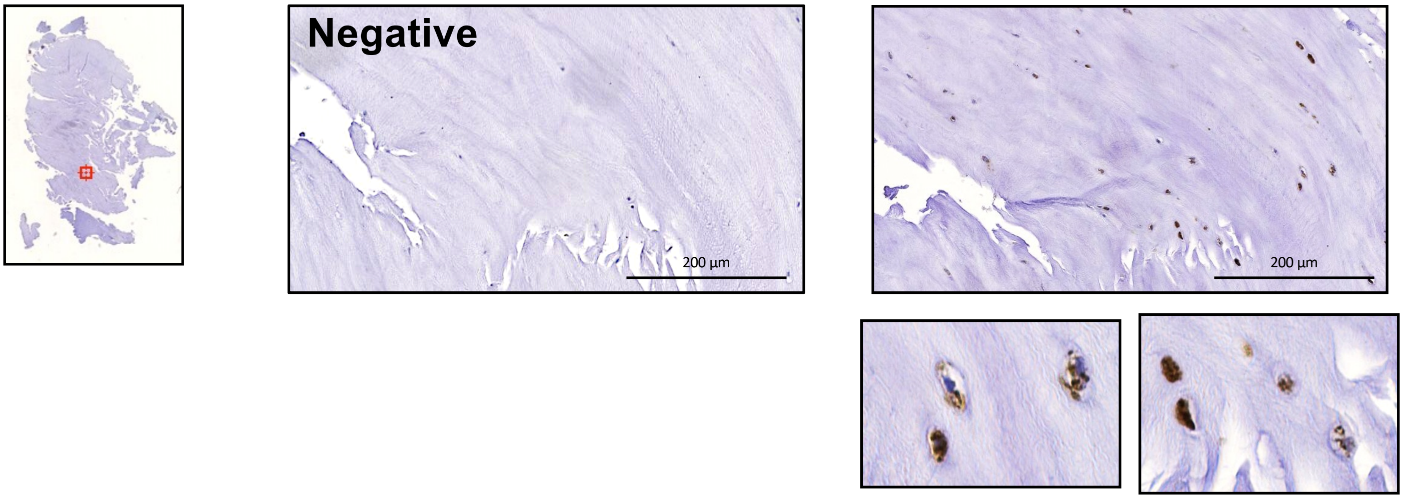

c. anti-β_6

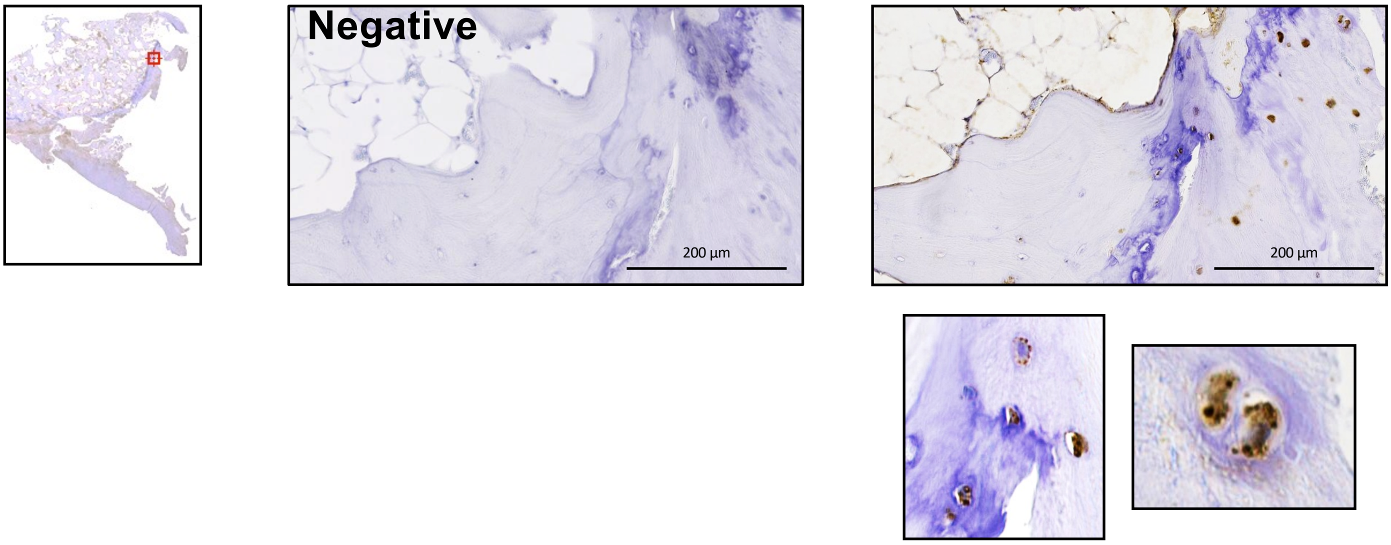

Figure S6. *In situ* punctate pattern for integrin staining
Representative IHC pictures of spinal osteophyte sections showing punctate pattern for **(a)** $\alpha_V\beta_5$ **(b)** $\alpha_V\beta_3$ and **(c)** β_6 staining. Red squares on IHC pictures indicate the location of zoomed areas (10x and 40x) on the whole osteophyte. Puncta are visible in smaller insert (40x). Scale bars are reported on each picture.
