# Supplementary material for: Toward diagnostic relevance of the αVβ5, αVβ3, and αVβ6 integrins in OA: expression within human cartilage and spinal osteophytes
Source: Bone Res. 2020 Sep 30;8:35. doi: 10.1038/s41413-020-00110-4 (PMC7527564; doi:10.1038/s41413-020-00110-4)
Supplement: Supplementary file 7 — Table S1 [file 41413_2020_110_MOESM7_ESM.pdf]

Patient #	Samples #	Osteophytes location	Diagnosis<br>(Medical imaging protocol)	Surgery
1	13	Lumbar<br>Posterior facet joint	Degenerative osteoarthritic changes in L4/L5	lumbar canal laminectomy
2	31	Lumbar<br>Posterior facet joint	Significant facet changes to L2-L3; facet osteoarthritis	lumbar canal laminectomy
3	5, 11, 15, 27, 33	Lumbar<br>Posterior facet joint	discarthrosis with severe canal stenosis in L3-L4 and moderate in L2-L3 and L4-L5	lumbar canal laminectomy
4	10, 14, 18, 34	Lumbar<br>Posterior facet joint	Bilateral posterior inter-apophyseal osteoarthritis	lumbar canal laminectomy
5	8, 9, 16, 26	Cervical<br>Anterior intervertebral disc	discarthrosis in C6-C7 with posterolateral and left foraminal disc protrusion	cervical arthrodesis
6	20, 23, 28	Cervical<br>Anterior intervertebral disc	C5-C6 and C6-C7 bilateral staged cervico-uncarthrosis	cervical arthrodesis
7	4, 7, 17, 25, 35	Lumbar<br>Posterior facet joint	Discopathy L2-L3, L3-L4 and L4-L5 with osteoarthritis of the posterior articular mass	lumbar canal laminectomy
8	6, 12, 19, 21, 22, 24, 30	Lumbar<br>Posterior facet joint	Advanced enlargements of osteoarthritis in L3-L4 and L4-L5 and posterior osteoarthritis changes in L5-S1	lumbar canal laminectomy
9	1, 2, 3, 29, 32	Cervical<br>Anterior intervertebral disc	Cervicarthrosis with foraminal stenosis in C5-C6 and C6-C7	cervical arthrodesis

Table S1. Osteophytes characterisation
35 spinal osteophytes were obtained from 9 patients. 23 osteophytes were collected from facet joints by lumbar canal laminectomy (6 patients) and 12 osteophytes were collected from intervertebral discs by cervical arthrodesis (3 patients).
