# Supplementary material for: Toward diagnostic relevance of the αVβ5, αVβ3, and αVβ6 integrins in OA: expression within human cartilage and spinal osteophytes
Source: Bone Res. 2020 Sep 30;8:35. doi: 10.1038/s41413-020-00110-4 (PMC7527564; doi:10.1038/s41413-020-00110-4)
Supplement: Supplementary file 9 — Table S3 [file 41413_2020_110_MOESM9_ESM.pdf]

$\alpha_v\beta_3$ integrin expression scores in spinal osteophytes								
Samples #	Cartilage + mCT area				Bone area			
	pure COL2	pure COL3	Overlapping COL2 COL3	Blood vessels	Blood vessels	Bone marrow cells	Bone lining cells	Osteocytes
1	irr	irr	3	3	irr	irr	irr	irr
2	irr	3	3	3	irr	irr	3	2
3	irr	irr	3	irr	irr	irr	3	0
4	irr	2	3	3	3	2	3	0
5	2	3	3	3	3	3	3	1
6	2	2	2	irr	3	irr	3	1
7	1	2	3	3	3	3	3	1
8	3	irr	irr	irr	3	3	3	1
9	0	irr	1	3	irr	2	3	1
10	1	1	2	3	3	irr	2	0
11	0	3	2	3	3	3	3	1
12	irr	3	3	irr	3	irr	3	1
13	irr	2	0	3	irr	irr	1	0
14	irr	3	3	irr	3	irr	3	1
15	2	3	2	3	3	3	3	1
16	2	irr	3	irr	3	3	3	1
17	irr	2	2	irr	3	irr	3	1
18	irr	3	3	irr	3	irr	3	2
19	irr	missing	3	irr	3	irr	3	1
20	irr	irr	3	3	3	3	3	0
21	irr	2	1	3	3	irr	3	2
22	irr	2	2	3	3	irr	3	1
23	irr	irr	2	3	irr	2	2	0
24	irr	3	3	irr	3	irr	3	1
25	irr	2	2	irr	3	irr	3	1
26	2	irr	3	3	irr	3	3	1
27	2	3	3	irr	irr	3	3	1
28	2	3	irr	irr	3	3	3	1
29	0	irr	2	irr	3	irr	3	0
30	irr	irr	3	irr	3	irr	3	1
31	irr	missing	1	irr	3	irr	3	1
32	irr	irr	2	irr	3	irr	3	0
33	0	irr	0	irr	3	irr	0	0
34	irr	3	3	irr	3	3	3	1
35	1	irr	2	irr	3	3	3	1
Mean vertical scores	1.3	2.5	2.3	3.0	3.0	2.8	2.8	0.8

Table S3. Scoring of $\alpha_v\beta_3$ integrin expression within human spinal osteophytes compartments

Expression score (from 0 to 3) attributed to $\alpha_v\beta_3$ integrin from immunostaining within each compartment or cell type of each osteophytes sample. 0: no cell stained; 1: <50% cells stained; 2: >50% cells stained and 3:all cells stained. Irr= irrelevant is indicated for compartment/cell type that are absent (*i.e.* 0 in **Table 1**) from all section analyzed. « missing » is indicated when the interest zone is found on all sections, except for the indicated staining. Last line of the table indicates mean vertical scores and corresponds to the mean of each osteophyte sample score for one compartment/cell type. Complementary, mean vertical score in function of compartment/cell types are presented as histograms in **Figure 4**.
